# Alphavirus-induced hyperactivation of PI3K/AKT directs pro-viral metabolic changes

**DOI:** 10.1371/journal.ppat.1006835

**Published:** 2018-01-29

**Authors:** Michela Mazzon, Cecilia Castro, Bastian Thaa, Lifeng Liu, Margit Mutso, Xiang Liu, Suresh Mahalingam, Julian L. Griffin, Mark Marsh, Gerald M. McInerney

**Affiliations:** 1 MRC Laboratory for Molecular Cell Biology, University College London, London, United Kingdom; 2 Department of Biochemistry, University of Cambridge, Cambridge, United Kingdom; 3 Cambridge Systems Biology Centre, University of Cambridge, Cambridge, United Kingdom; 4 Department of Microbiology, Tumor and Cell Biology (MTC), Karolinska Institutet, Stockholm, SE, Sweden; 5 Institute of Virology, Faculty of Veterinary Medicine, University of Leipzig, Leipzig, Germany; 6 Institute of Glycomics, Griffith University, Gold Coast, Queensland, Australia; University of Washington, UNITED STATES

## Abstract

Virus reprogramming of cellular metabolism is recognised as a critical determinant for viral growth. While most viruses appear to activate central energy metabolism, different viruses have been shown to rely on alternative mechanisms of metabolic activation. Whether related viruses exploit conserved mechanisms and induce similar metabolic changes is currently unclear. In this work we investigate how two alphaviruses, Semliki Forest virus and Ross River virus, reprogram host metabolism and define the molecular mechanisms responsible. We demonstrate that in both cases the presence of a YXXM motif in the viral protein nsP3 is necessary for binding to the PI3K regulatory subunit p85 and for activating AKT. This leads to an increase in glucose metabolism towards the synthesis of fatty acids, although additional mechanisms of metabolic activation appear to be involved in Ross River virus infection. Importantly, a Ross River virus mutant that fails to activate AKT has an attenuated phenotype *in vivo*, suggesting that viral activation of PI3K/AKT contributes to virulence and disease.

## Author summary

In order to replicate to high titres, viruses need a sufficient supply of building blocks from the cell in the form of amino acids, nucleotides and lipids. To this end, viruses have evolved different mechanisms to reprogram and exploit host metabolism towards the synthesis of new biomass. Pharmacological inhibition of pathways involved in metabolic activation can represent an opportunity for therapeutic intervention. In this study, we explore how members of the alphavirus group, including Semliki Forest virus (SFV) and Ross River virus (RRV), modify host metabolism. We discover that both viruses activate cellular glycolysis by activating the PI3K/AKT pathway. This activation is mediated by a YXXM motif in the viral protein nsP3, which binds to PI3K and initiates AKT signalling. In SFV, mutations in this motif prevent AKT activation, reduce virus-induced metabolic activity, and lower viral replication. In the human pathogen RRV the same mutation causes less severe disease *in vivo*. We therefore conclude that AKT activation during infection with these viruses is an important determinant of pathogenicity.

## Introduction

Many cellular functions have evolved in close interdependence with cell metabolism and nutrient availability. The immune system itself, central for host defence from pathogens, is highly dependent on the metabolic activity of the organism for critical processes such as cell proliferation and differentiation [1]. Importantly, many viral pathogens significantly alter host cell metabolism in order to fulfil their energy requirements, but their impact on metabolic balance has only recently begun to be investigated. The emerging picture suggests that viruses use a range of strategies to activate glycolysis [2–6] and modulate glutamine metabolism [7–9]. However it is less clear how viral proteins drive metabolic changes and whether these mechanisms are conserved across related viruses. Understanding the effect of virus replication on cellular metabolism is therefore needed not only to define virus requirements, but also to understand the impact of viral replication on the energy status of an infected host, and its role in pathogenesis.

Alphaviruses are enveloped, positive sense, single stranded RNA viruses, transmitted to humans and a variety of mammals by insects, often mosquitos. There is currently no available vaccine or treatment for most alphaviruses and this contributes to their rapid spread and to several recent epidemics [10]. Causing approximately 5000 cases each year, Ross River virus (RRV) is the most prominent human alphavirus in the South Pacific region, responsible for a debilitating musculoskeletal disease [11]. The link between infection and pathogenicity remains unclear. In contrast, Semliki Forest virus (SFV) generally does not cause disease in humans, but is neuropathogenic in mice and has often been used as a model system for the study of alphavirus biology [12]. In this study, we apply nuclear magnetic resonance spectroscopy (NMR) and gas chromatography-mass spectrometry (GC-MS) to analyse the metabolic alterations that accompany cellular infection with SFV and RRV. In SFV infected cells we measured sustained activation of glycolysis and of the pentose phosphate pathway (PPP) towards higher synthesis of new metabolites, and demonstrated that this is dependent on virus-induced activation of PI3K/AKT via a YXXM motif in the viral non-structural protein (nsP) 3. To test whether the presence of this motif is sufficient to predict changes in host metabolism during alphavirus infection, we examined the metabolic profile of cells infected with RRV, an alphavirus carrying the same motif in nsP3, whose metabolic profile has never been characterised. Although infection with RRV also triggers additional mechanisms of metabolic activation, disruption of the YXXM motif causes lower viraemia and reduced pathogenicity *in vivo*, thus demonstrating the importance of AKT activation in viral pathogenesis.

## Results

### SFV infection of SH-SY5Y increases glucose metabolism

**Fig 1:**
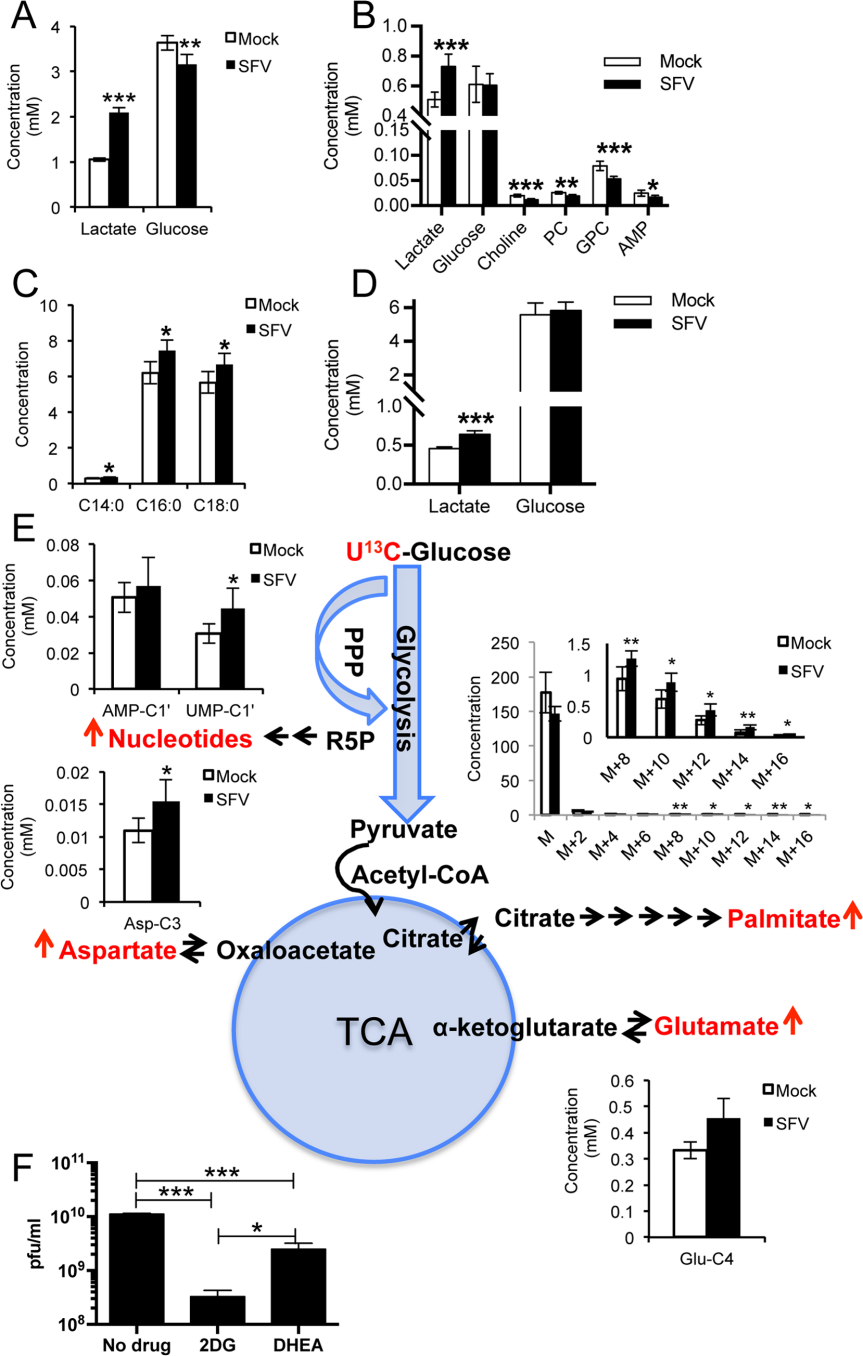
SFV infection activates glucose metabolism and increases fatty acid levels. Concentrations (mM) of **A.** lactate and glucose in the media and **B.** selected metabolites in the cells following infection with SFV. **C.** Levels of selected cellular fatty acids measured by GC-MS. **D.** Concentrations (mM) of lactate and glucose in the media of primary rat cortical neurons (MOI 5, 8 hpi). **E.** Schematic showing the metabolic products of increased glucose consumption. Metabolites directly measured by NMR spectroscopy or GC-MS are in red. For the aqueous metabolites, each plot represents the concentration (mM) of the indicated resonance, as calculated from the volumes of HSQC spectra normalised to formate, added as an internal standard to samples. The concentration of palmitate was calculated from the intensity of the single isotopic mass normalised to the internal standard. Six samples per group were analysed. Data are presented as means ± SD. **F.** Virion release at 16 hpi after treatment with inhibitors of glycolysis (2DG) or PPP (DHEA) or mock treated. Drugs were administered at 25 mM and 100 μM, respectively, at the same time as SFV infection. Data are presented as means ± SEM. T-tests (with Bonferroni corrections when the comparison involved more than two groups) were performed. * 0.05> p < 0.01; ** 0.01> p < 0.001; *** p < 0.001.

SFV has been used extensively as a model to study the cell biology of alphavirus infection. Although SFV infects a variety of cells in tissue culture, neurons are key targets *in vivo* [12]. Therefore we chose to study the metabolic alterations induced by SFV in the neuroblastoma cell line SH-SY5Y, a well-known model for neuronal function and differentiation [13], after 5 days differentiation with *trans*-retinoic acid (**S1A Fig**). In contrast to rapidly dividing cells, these post-mitotic neurons better reflect the less metabolically active environment that SFV would encounter *in vivo*, and therefore represent a good model to study how SFV infection might alter cell metabolism. Metabolic changes were profiled by ^1^H-NMR and GC-MS at 8 hours post infection (hpi). The complete list of assigned metabolites is shown in **S1 Table** (^1^H-NMR) and **S2 Table** (GC-MS). SFV infection doubled lactate concentration in the media (p = 1.1*10^−6^) and increased it by 43% in the cells (p = 3.97*10^−4^) (**Fig 1A** and **1B**), while it decreased glucose concentration in the media (**Fig 1A**) by 10% (p = 1.93*10^−3^) and reduced choline, phosphocholine (PS), glycerophosphocholine (GPC), and adenosine monophosphate (AMP, an intermediate of nucleotide metabolism) levels in the cells (**Fig 1B**). It also markedly increased palmitic (C16:0) and stearic (C18:0) acids (**Fig 1C**). These data suggest increased glycolytic activity and fatty acid synthesis upon SFV infection. An increase by 40% in lactate accumulation (p = 3.98*10^−4^) was also observed in the media of infected primary rat cortical neurons **(Fig 1D)**, validating the SH-SY5Y model. The kinetics of this metabolic reorganisation mirrored the kinetics of viral protein expression **(S1B Fig)**, with a progressive accumulation of lactate with time and a simultaneous decrease of glucose in the media and glycerophosphocholine in the cells (**S1C and S1D Fig**).

To understand the consequences of this increased glycolytic activity, we followed the fate of uniformly labelled ^13^C glucose ([U-^13^C]glucose) 8 h after SFV infection. In infected cells we observed increased concentrations of [4-^13^C]glutamate (p = 0.02), [3-^13^C]aspartate (p = 0.03), and labelled palmitate (**Fig 1E**), suggesting an increased flux of glucose through the TCA cycle and a consequent increased export of citrate to the cytoplasm for *de novo* synthesis of fatty acids. No changes in the total level of succinate (**S1E Fig**) were found in control unlabelled samples, suggesting no accumulation of this intermediate during infection. Increased concentrations of labelled UMP (p = 0.04) and the constant levels of labelled AMP (**Fig 1E**), together with the marked decrease in the total concentration of this nucleotide in unlabelled samples (**S1E Fig**), suggest increased synthesis (and use) of nucleotides, supporting a key role for the PPP.

The glucose analogue 2-deoxyglucose (2DG), an inhibitor of glycolysis, has been shown to decrease SFV replication when added 16 h before infection [14]; however to confirm the importance of glycolysis and the PPP during the course of viral replication only, we treated cells with 2DG or the glucose-6-phosphate dehydrogenase inhibitor (dehydroepiandrosterone, DHEA, an inhibitor of the PPP) at the same time of infection with SFV. At 16 hpi, 2DG reduced production of new infectious virions by almost 2 logs, and DHEA by almost 1 log (**Fig 1F**). Profiling of SH-SY5Y cells treated for 16 hours with either inhibitor in the absence of infection showed the anticipated effects on glycolytic metabolites (**S1G Fig**). In both cases, AMP concentrations were significantly reduced (p = 0.001 for 2DG and for DHEA). In cells treated with 2DG (which replaces glucose in the first step of glycolysis), glucose was present at higher concentration in both media and cells, while a significant reduction in lactate production was observed. In cells treated with DHEA (which inhibits glucose entrance into the PPP), we observed an increased consumption of glucose in both media and cells and a simultaneous increase in lactate production, likely a compensatory effect triggered by the PPP inhibition. During infection, inhibition of the first step of glycolysis with 2DG, which is detrimental for both glycolysis and PPP, had a more dramatic effect on virus production (**Fig 1F**). Importantly, no significant toxicity was observed following treatment with either drug (**S1F Fig**), and no effect was observed on the early stages of viral replication (**S1H Fig**), indicating that blocking glycolysis or the PPP does not affect SFV infectivity.

### SFV increases glycolysis by activating the PI3K/AKT signalling pathway

**Fig 2:**
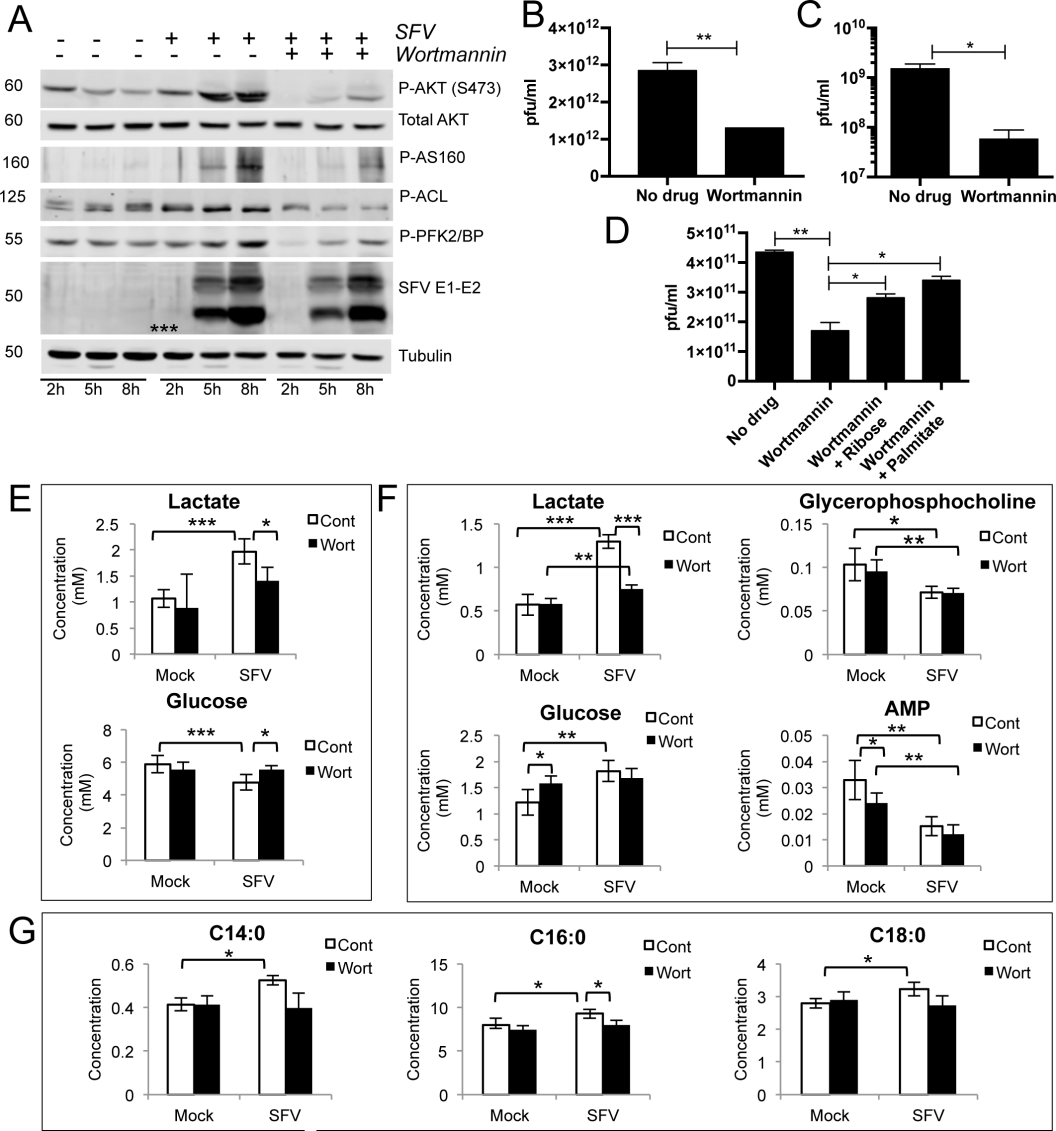
The PI3K inhibitor Wortmannin antagonises SFV-induced AKT activation, glycolysis, and production of new virions. **A.** Kinetics of activation of AKT and downstream targets in SH-SY5Y cells infected with SFV at MOI 5, in the presence or absence of 10 μM Wortmannin, administered at the same time as SFV infection. Synthesis of new virions from SFV-infected SH-SY5Y **(B)** or rat primary cortical neurons **(C)** after treatment with 10 μM Wortmannin, administered at the same time as SFV infection (MOI 3). After 16h, virions in the supernatant were quantified by plaque assay. **D.** Synthesis of new virions from SFV-infected SH-SY5Y after treatment with Wortmannin alone, or together with 40 mM ribose or 400 μM BSA-conjugated palmitate, administered at the same time as SFV infection (MOI 3). After 16h, virions in the supernatant were quantified by plaque assay. Data are presented as means ± SEM. Concentrations (in mM) of **E.** lactate and glucose in the media, and **F.** lactate, glucose, glycerophosphocholine and AMP in the cells, in mock- or SFV-infected samples treated with Wortmannin or DMSO control (8 hpi). **G.** Levels of selected cellular fatty acids in mock- or SFV-infected cells treated with Wortmannin or DMSO. Six samples per group were analysed. Data are presented as means ± SD. Statistics as in Fig 1. ANOVA was performed when comparison included more than two groups.

The extensive and rapid increase in glycolysis upon SFV infection is reminiscent of the dramatic metabolic reprogramming typical of cancer cells [15], suggesting that SFV might activate a metabolic “master switch”, able to rapidly reprogram cellular metabolism. The PI3K/AKT signalling pathway has been shown to be activated upon SFV infection in a very strong and sustained manner, here referred to as “hyperactivation”. This PI3K/AKT hyperactivation overrides inhibition by growth factor depletion and requires the viral protein nsP3 [16,17]. However, no link to metabolism was made in these previous studies. PI3K/AKT hyperactivation in SH-SY5Y cells upon SFV infection was analysed by western blot at various times post infection. Mirroring the kinetics of viral replication and increased glycolysis, we observed phosphorylation of AKT from 5 hpi in the SH-SY5Y cells, with even higher levels at 8 hpi **(Fig 2A)**. In agreement with a role for PI3K/AKT regulation of cell metabolism, we also observed phosphorylation of the downstream targets phosphofructokinase 2 (PFK2), the Rab GTPase-activating protein AS160, which increases trafficking of glucose transporters to the plasma membrane, and ATP citrate lyase (ACL), the enzyme responsible for cytosolic acetyl-coA synthesis from citrate. Phosphorylation of AKT was also observed in primary rat cortical neurons (**S2A Fig** and **S2B Fig**). Consistent with the activation of AKT, a kinase that modulates glycolysis primarily by phosphorylating key glycolytic enzymes, we did not observe any increase in the mRNA levels of glycolytic genes (**S2C Fig**).

The PI3K inhibitor Wortmannin almost entirely abrogated infection-mediated phosphorylation of AKT and of downstream targets without substantially changing the expression of SFV E1-E2 proteins (**Fig 2A**), indicating that reduced AKT activation is not due to reduced infection. No inhibition was detected upon Wortmannin treatment at 8 hpi (**S2E Fig**), suggesting that viral entry, early RNA replication and protein synthesis are not affected. Conversely, Wortmannin decreased the release of progeny virus by ~60% compared with the untreated control (**Fig 2B**), implying that activation of AKT signalling is more important for late stages of viral replication. Indeed, adding Wortmannin either at the same time or two hours after virus infection resulted in similar levels of viral decrease (**S2F Fig**). An even more striking decrease was observed in primary neurons (**Fig 2C**). Similar levels of inhibition were measured using a different PI3K inhibitor, LY294002 (**S2G Fig**). Cell viability was not markedly compromised by 16 h treatment with either drug (**S2D Fig**).

We next tested whether Wortmannin affected SFV-induced glycolysis. In mock-infected samples, Wortmannin caused a marginal reduction of cellular glycolytic activity and did not affect myristic, palmitic or stearic acid content. In SFV infected cells, Wortmannin induced a significant decrease in lactate levels in both media (by 40%, p = 0.027) and cells (by 73%, p = 0.003) (**Fig 2E** and **2F**, respectively), a decreased use of glucose (by 13%, p = 0.013) (**Fig 2E**), and reduced fatty acids levels (**Fig 2G**). The dramatic decrease of glycolysis in infected cells treated with Wortmannin suggests that PI3K/AKT is indeed responsible for activating this pathway upon SFV infection, and for the increase in glycolytic products and fatty acid synthesis. Production of new infectious virions, inhibited by Wortmannin, could be rescued by 25% by providing ribose, a nucleotide precursor generated primarily through the PPP branch of glycolysis, and by 35% by palmitate, a precursor for the more complex lipids generated from glycolysis (**Fig 2D**). This suggests that, although other factors are also likely to restrict SFV replication upon Wortmannin treatment, activation of glycolysis is important for the generation of extra metabolic building blocks, needed for maximal production of new virus.

### Y369 in SFV nsP3 is necessary for AKT activation and binding of nsP3 to the SH2 domain of p85

**Fig 3:**
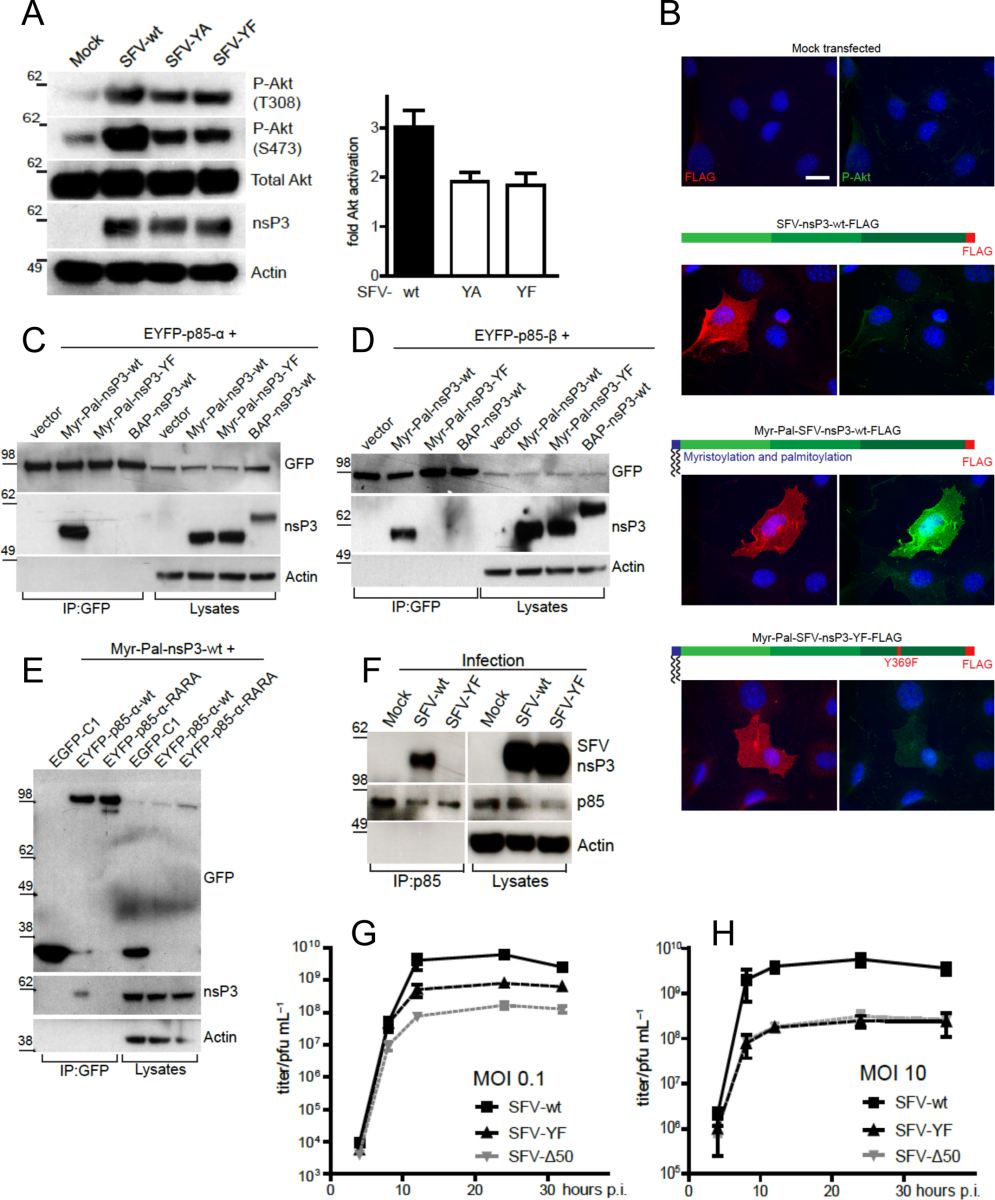
The YXXM motif in SFV nsP3 mediates PI3K/AKT hyperactivation. **A.** AKT activation in BHK cells infected at MOI 10 as indicated and lysed at 8 hpi, together with densitometry of phosphorylated AKT (S473), normalised to total AKT signals and relative to mock-infected cells (mean of three independent experiments ± SEM). **B.** Immunofluorescence showing activation of AKT (S473, green) in MEFs transfected for 24 h with the indicated DNA plasmids (red: FLAG-nsP3, blue: nuclei). Representative epifluorescence images are shown. Scale bar: 20 μm. Co-immunoprecipitations showing the interaction between EYFP-p85−α (**C** and **E**) or EYFP-p85−β (**D**) in HEK293T cells transfected with the indicated constructs for 24 h. EYFP-tagged p85 constructs were immunoprecipitated with an anti-GFP antibody. BAP: biotin acceptor peptide, Myr-Pal: myristoylation and palmitoylation. **F.** Interaction of endogenous p85 and viral nsP3 in lysates of BHK cells infected at MOI 10 for 8 h, after immunoprecipitation with an anti-p85 antibody. Growth curves of SFV-wt and SFV-YF in BHK cells infected at MOI 0.1 (**G**) or 10 (**H**) for the indicated times. Virus titres at each time point were determined by plaque assay. Data show the mean ± SEM of three experiments.

We next tried to understand how SFV nsP3 activates PI3K by searching for the presence of eukaryotic linear interaction motifs in this protein. Using the database ELM (http://elm.eu.org/), we identified the well-characterised PI3K activation motif YXXM in the amino acid stretch Y369-E370-P371-M372, situated in the C-terminal hypervariable domain (**S3A Fig**). To test whether this motif is relevant for PI3K/AKT activation, we generated viral mutants where Y369 was replaced with an alanine (SFV-YA) or with a phenylalanine (SFV-YF). In BHK cells, both viruses, although replicating in a similar manner as indicated by nsP3 levels, failed to activate AKT to the same extent as the wild type virus, confirming the importance of this motif in PI3K/AKT activation (**Fig 3A**). Consistent with low PI3K/AKT activation, the viral replication complexes (RC) of both mutant viruses were mainly localised at the cell periphery, while wt SFV showed efficient RC internalisation (**S3B Fig**), as previously reported [16]. Disruption of the YEPM sequence by leaving the tyrosine unchanged and mutating the three downstream residues to alanines (SFV-YAAA) also reduced AKT phosphorylation (**S3C Fig**), indicating that a complete YXXM motif is required for AKT activation. To minimise potential unrelated structural changes, SFV-YF was selected for further studies. Anchorage of nsP3 to the plasma membrane is required for PI3K/AKT activation, however membrane anchored Myr-Pal-tagged nsP3-YF failed to activate AKT, further confirming the relevance of the YXXM motif for activation of the pathway (**Fig 3B**).

Activation of class IA PI3Ks is typically mediated through the interaction between the phosphorylated tyrosine in a YXXM motif and the arginines in the FLVRD/E motifs of the SH2 domains of the p85 subunit of PI3K [18]. We therefore hypothesized that the SFV-nsP3 YXXM motif binds the SH2 domain of p85, and that either mutation at Y369 in nsP3 or mutations in the SH2 domains of p85 would abolish the interaction. Consistent with this scenario, Myr-Pal-nsP3-wt, but not Myr-Pal-nsP3-YF or biotin acceptor peptide (BAP)-tagged nsP3-wt without a membrane anchor, was pulled-down by EYFP tagged p85α (EYFP-p85-α) (**Fig 3C**) and by EYFP tagged p85β (EYFP-p85-β) (**Fig 3D**). Equally, nsP3 co-immunoprecipitated with wild type EYFP tagged p85 (EYFP-p85-α-wt), but not with a mutated form of p85 where the arginines in the FLVRD/E motifs in both SH2 domains were mutated to alanines (**Fig 3E**) (“RARA mutant”) [19,20]. Importantly, we also show by co-immunoprecipitation that nsP3-wt but not nsP3-YF interacts with endogenous p85 in the context of SFV infection (**Fig 3F**).

The effect of the Y369F mutation in nsP3 on viral replication was tested by measuring viral release over time after infection of BHK cells at low (0.1) and high (10) MOI, compared to wt SFV and SFV-Δ50 carrying a larger deletion in nsP3, also affecting PI3K activation (**S3C Fig**). Consistent with a role for PI3K/AKT activation on late stages of the virus life cycle, viral titres were similar at early times post-infection but differed at later time points, with wt SFV titres exceeding SFV-YF by approximately one (MOI 0.1; **Fig 3G**) or more (MOI 10; **Fig 3H**) orders of magnitude by 12 hpi.

Taken together, these data show that during infection, the YXXM motif of SFV nsP3 binds the SH2 domains of p85 at the plasma membrane and activates the PI3K/AKT pathway leading to internalisation of replication complexes and efficient virus replication (**S3D Fig**).

### The Y369F mutation abolishes the metabolic changes induced by SFV infection

**Fig 4:**
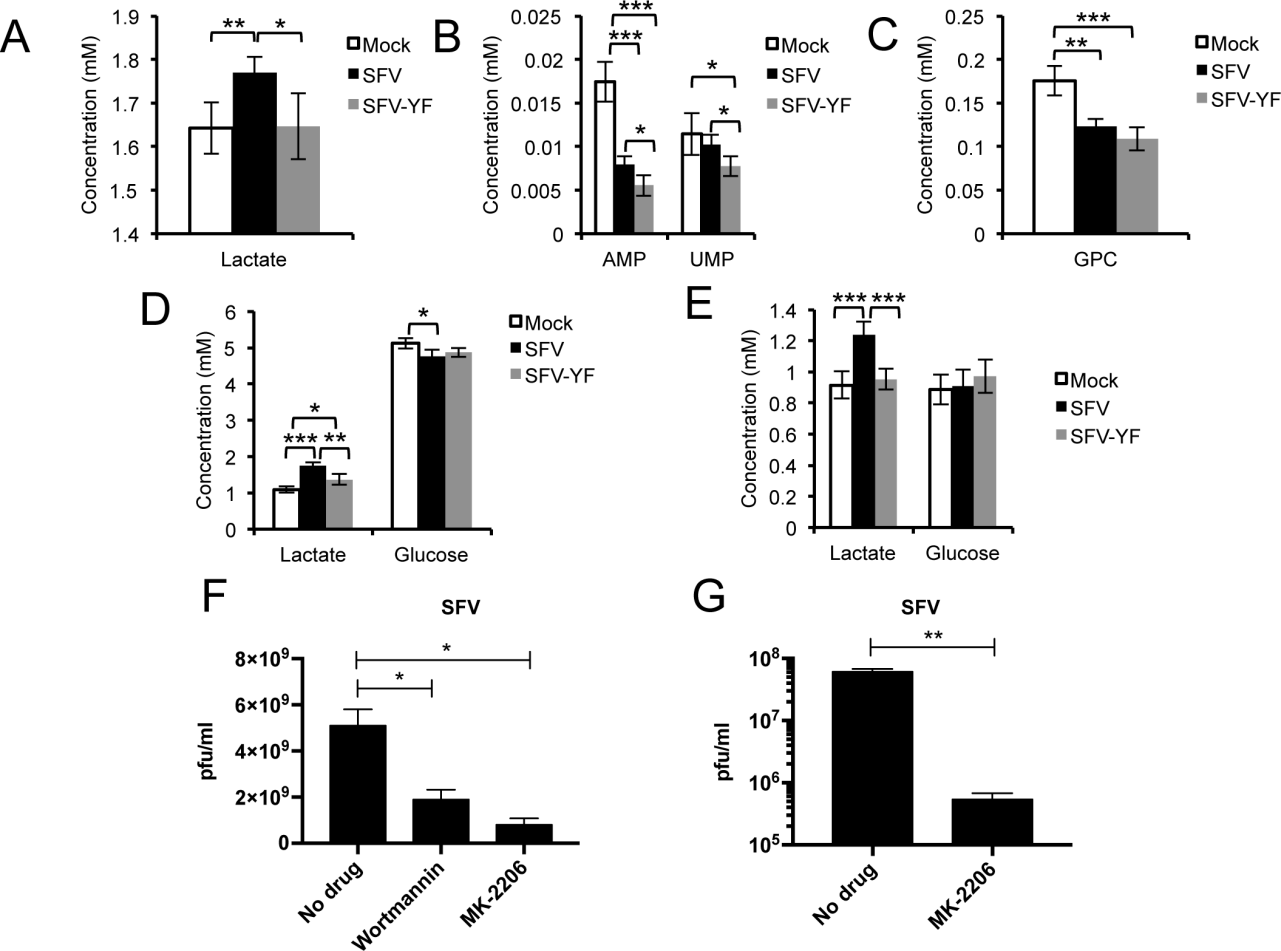
SFV-YF fails to activate AKT and does not increase glucose metabolism. Concentrations (mM) of **A.** lactate in the media, and **B.** AMP and UMP and **C.** glycerophosphocholine in the cells of samples mock-, WT SFV- or SFV-YF-infected at MOI 1 (8 hpi). Concentrations (in mM) of lactate and glucose in the media (**D**) and in the cells (**E**) of samples mock-, WT SFV- or SFV-YF-infected at MOI 5 (8 hpi). Six samples per group were analysed. Data are presented as means ± SD. SFV release of new virus particles upon 8h treatment with Wortmannin (2 μM) and MK-2206 (7 μM) in differentiated SH-SY5Y (**F**) and MK-2206 (7 μM) in primary rat cortical neurons (**G**). Data are presented as means ± SEM. Statistics as in Fig 1. ANOVA was performed when comparisons included more than two groups.

To establish the role of nsP3 Y369 in reprogramming cell metabolism, we compared the metabolic profiles of SH-SY5Y upon mock infection or infection with either wt SFV or SFV-YF. The inability of SFV-YF to activate AKT and its downstream targets over the course of infection was confirmed in SH-SY5Y (**S4A Fig**). In addition, the production of new viral progeny was lower after infection with SFV-YF compared to wt virus (**S4B Fig**). After 8 hours of infection at MOI 1, lactate levels in the media were higher for wt SFV compared to both mock controls and SFV-YF (**Fig 4A**), suggesting activation of the glycolytic pathway in cells infected with the wt virus, but not the mutant. As observed previously, AMP was decreased in the wt SFV infected samples compared to mock controls, but a larger decrease was seen in the SFV-YF infected cells. UMP did not differ between samples infected with wt SFV and mock controls, but was significantly decreased in SFV-YF, suggesting the possibility that in samples infected with SFV-YF nucleotide usage exceeded new synthesis (**Fig 4B**). Finally, a similar decrease in glycerophosphocholine levels between wt SFV and SFV-YF samples (**Fig 4C**) showed that the mutation only affected the activation of the glycolytic pathway.

With higher viral load (MOI 5), increased levels of lactate were detected at 8 hpi both in the media (**Fig 4D**) and in cells (**Fig 4E**) infected with wt SFV compared to mock and SFV-YF (by 27% in the media, p = 0.0046, and by 29% in the cells, p = 0.00012), with lower concentrations of glucose in the media compared to the mutant (by 2%, p = 0.017). These results also suggest an important contribution of the TCA cycle: SFV-YF-infected cells contained significantly lower concentrations of glutamate (a source of 2-oxoglutarate) and succinate (**S4C Fig** and **S4D Fig**), suggesting depletion of these TCA intermediates, presumably as citrate is consumed to feed fatty acid synthesis, as suggested by the labelling data (**Fig 1E**). Overall, these data mirror the results obtained following treatment with Wortmannin, and emphasise the crucial role of the YXXM motif in SFV nsP3 in the hyperactivation of AKT and in host cell metabolic reprogramming.

SFV-induced PI3K/AKT hyperactivation likely affects other downstream features in addition to cell metabolism. We assessed one such feature, the mammalian target of rapamycin (mTOR), involved in translational control, using phosphorylation of rpS6 as a readout. Indeed, mTOR was activated in SFV-wt infected cells even under nutrient and growth factor depletion, which was not the case in SFV-YF or mock infected cells **(S4F Fig)**. However, despite sustained activation of mTOR, treatment of cells with rapamycin to specifically inhibit the SFV-induced mTOR hyperactivation had no major effect on wt SFV growth kinetics (**S4G Fig**). We hence conclude that the activation of mTOR seen during WT SFV infection as a downstream effect of infection-induced PI3K/AKT hyperactivation is not critical for virus growth *in vitro*.

### Pharmacological inhibition of AKT reduces SFV replication

AKT inhibitors have been studied as anticancer treatments for nearly two decades, resulting in the development of a number of compounds currently undergoing clinical trials [21]. MK-2206 is a diphenylquinoxaline analogue that inhibits AKT by locking the kinase in a closed conformation and it has been tested alone or in combination against several malignancies [22,23]. Treatment of differentiated SH-SY5Y with MK-2206 caused a reduced release of infectious virions even more striking than Wortmannin (~84% vs. ~63%, respectively) (**Fig 4F**). As for Wortmannin and LY294002, we observed an even more dramatic inhibition in rat primary neurons (~2 logs, **Fig 4G**). Cell viability following MK-2206 treatment is shown in **S4H Fig**.

### The alphavirus Sindbis virus activates glycolysis in a PI3K/AKT independent manner

**Fig 5:**
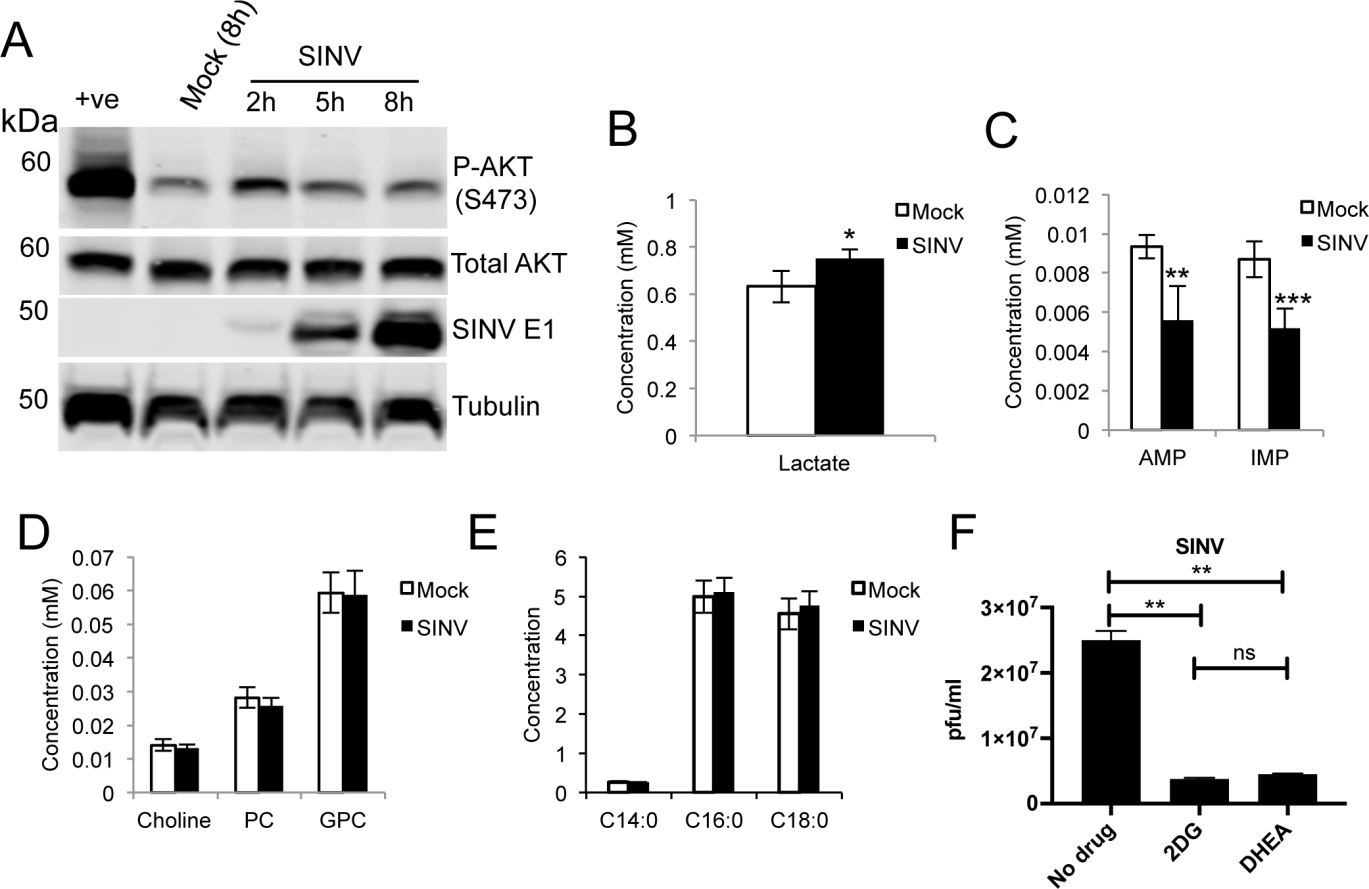
SINV does not activate AKT and displays a metabolic profile different from SFV. **A.** Kinetics of AKT activation upon infection of differentiated SH-SY5Y cells with SINV. The positive control (+ve) was obtained by 20 mins treatment with 200 μM hydrogen peroxide and 100 μM sodium orthovanadate. Intracellular levels of lactate (**B**), nucleotides (**C**), cholines (**D**), and fatty acids (**E**) in cells infected with SINV for 8h. Data are presented as means ± SD. Six samples per group were analysed. **F.** Virion release at 16 hpi after treatment with inhibitors of glycolysis (2DG) or PPP (DHEA) or mock treated. Drugs were administered at 25 mM and 100 μM, respectively, at the same time as SINV infection. Data are presented as means ± SEM. Statistics as in Fig 1.

Interestingly, Sindbis virus (SINV), an alphavirus lacking the YXXM motif in nsP3, failed to induce sustained AKT activation (**Fig 5A**) and only caused a ~30% increase in glycolytic activity (measured as lactate over glucose ratio (**Fig 5B**)), compared to the ~100% increase observed for SFV. Importantly, while we measured a decrease in nucleotide concentrations (**Fig 5C**), no decrease in choline and phosphocholine levels was observed upon SINV infection (**Fig 5D**) and no changes were measured in the concentrations of myristic, palmitic or stearic acids in infected cells (**Fig 5E**). Thus SINV, which does not rely on AKT hyperactivation during replication, may have evolved different mechanisms of metabolic activation and lipid use/synthesis. Inhibition of glycolysis (with 2DG) or of the PPP (with DHEA) caused similar degrees of inhibition of almost 1 log, suggesting that SINV relies on these two metabolic pathways (**Fig 5F**).

### Mutation of the YXXM motif in Ross River virus leads to an attenuated phenotype *in vivo*

As indicated above, the sequence YXXM is not present in the nsP3 of all alphaviruses. Systematic sequence analysis however reveals the presence of YXXM in nsP3 of the human alphavirus pathogen Ross River virus (RRV), located in a similar position as in SFV nsP3, despite overall low conservation of the nsP3 hypervariable domain between the two viruses (**S5A Fig**).

**Fig 6:**
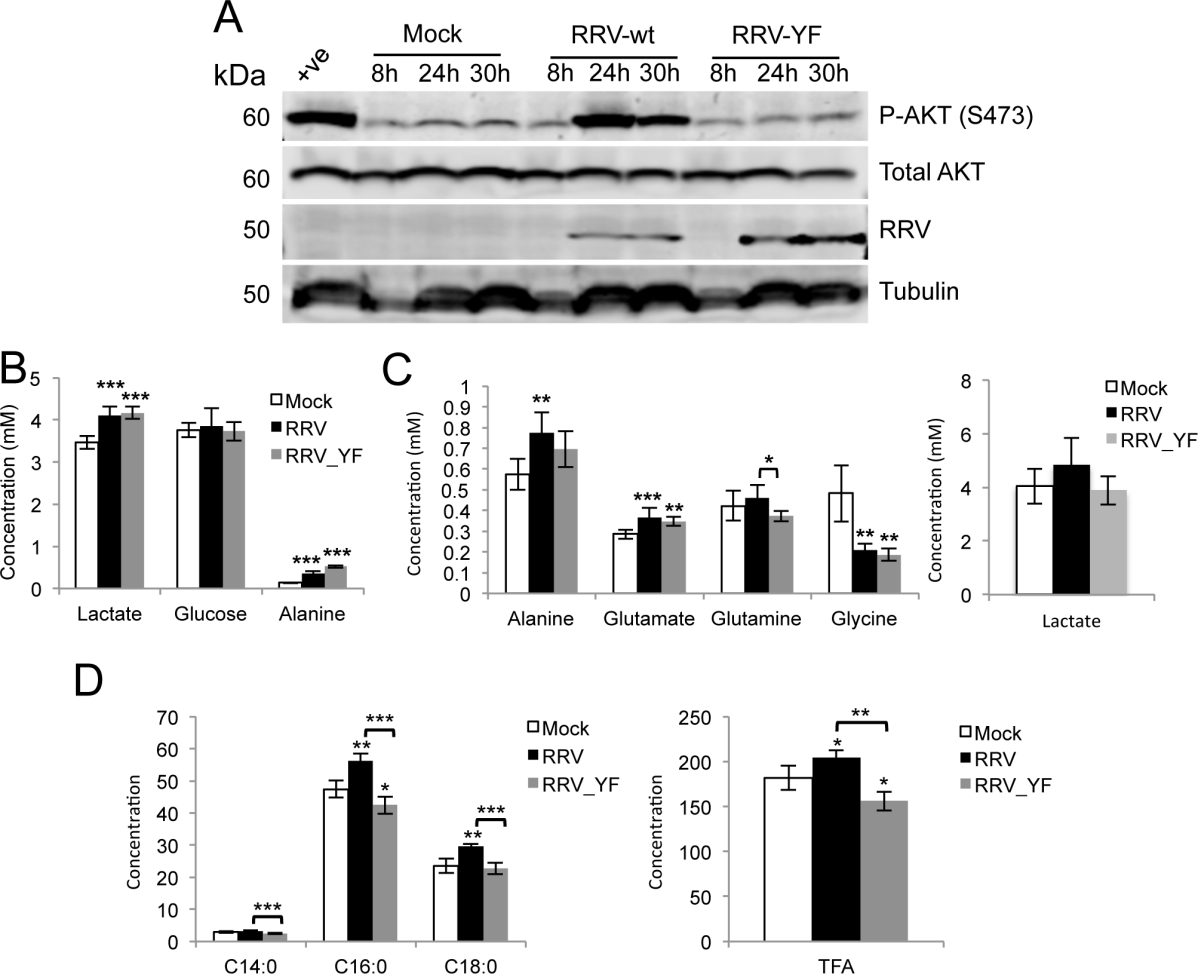
RRV activates AKT and increases glucose and glutamine metabolism *in vitro*. **A.** AKT activation in differentiated C2C12 cells infected with RRV-wt or RRV-YF at MOI 5 and lysed at 8, 24, and 30 hpi. Positive control as in Fig 5. Metabolite concentrations in the media (**B**) and in the cells (**C**) of samples mock-, RRV-wt or RRV-YF infected at MOI 5 (24 hpi). **D.** Fatty acids concentrations in the same samples as in B. TFA = Total Fatty Acid. Data are presented as means ± SD. Six samples per group were analysed. Statistics as in Fig 1. ANOVA was performed when comparisons included more than two groups.

As with SFV, wild type RRV (RRV-wt) induced hyperactivation of AKT both in BHK cells (**S5B Fig**) and in differentiated C2C12 (**Fig 6A**), a murine cell line commonly used as a model of muscle myotubes, a RRV target *in vivo*. RRV in which the relevant tyrosine (Y356) was mutated to phenylalanine (RRV-YF) induced lower levels of AKT activation (**Fig 6A** and **S5B Fig**) and prevented internalisation of RC (**S5C Fig**). However, when we measured release of RRV-wt and -YF in BHK cells (**S5D Fig,** left and middle panels), no significant differences were observed between the two viruses, neither in a multi-step (MOI 0.1) nor a single-step growth curve (MOI 5). Similar results were obtained in C2C12 cells (MOI 5, **S5D Fig,** right panel). To understand whether this lack of difference could be explained by a metabolic phenotype somewhat different from the one observed for SFV, we examined the metabolic profile of differentiated C2C12 infected with RRV-wt or RRV-YF. Metabolic analysis of RRV-wt infected cells showed activation of glycolysis with an 18% increase in lactate in the media (p = 0.0008), and higher levels of alanine in media and cells (**Fig 6B** and **6C**, respectively). Interestingly, no significant decrease in glycolysis was measured in RRV-YF infected cells compared to RRV-wt, as we also found a 20% increase in lactate levels in the media (p = 0.00001, compared to mock) and higher concentrations of alanine in cells infected with RRV-YF (**Fig 6B** and **6C**). However, significantly higher levels of fatty acids were measured upon infection with RRV-wt than with RRV-YF (**Fig 6D**). The concomitant differences in glutamine and glutamate levels in cells infected with either virus (**Fig 6C**), which were not observed upon infection with SFV (**S4C Fig** and **S4E Fig**), suggest the possibility that RRV may rely on an additional, AKT-independent mechanism of metabolic activation that stimulates both glucose and glutamine metabolism. Despite the differences between SFV and RRV, activation of AKT by either virus was linked to increased fatty acid synthesis.

**Fig 7:**
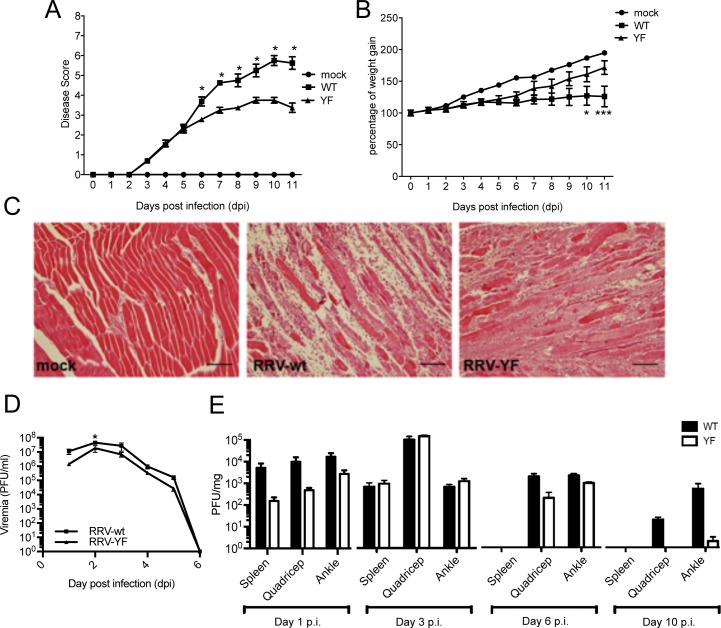
RRV-YF is attenuated *in vivo*. Disease scores (**A**) and weights (**B**) of twenty day old C57BL/6 wt mice injected subcutaneously with 10^4^ pfu of RRV-wt or RRV-YF or mock-infected with PBS. **C.** Representative histology images (hematoxylin-eosin staining) of quadriceps at 10 days p.i. Scale bar: 100 μm Virus titres from tail blood (**D**), spleen, quadricep, and ankle (**E**) at different time points after infection, determined by plaque assay. Data are presented as mean ± SEM of 3–4 mice per group. Statistical significance for the difference between RRV-wt- and RRV-YF-infected mice was determined using two-way ANOVA with Bonferroni post-test (B, D, and E) or non-parametric Mann–Whitney test (A). 0.05> p < 0.01; ** 0.01> p < 0.001; *** p < 0.001.

We next tested whether hyperactivation of the AKT pathway by RRV-wt but not RRV-YF resulted in differences in pathogenesis *in vivo* in a murine model of infection. C57BL/6 mice were subcutaneously infected with 10^4^ pfu of RRV-wt or RRV-YF and monitored daily for disease signs. Limb weakness and loss of gripping ability, which in RRV-wt infected mice reached a disease score of 5.5 on day 10, were significantly milder in mice infected with RRV-YF, with disease scores not exceeding 4 (**Fig 7A**). Also, the average weight gain of RRV-wt infected mice was considerably lower than mice infected with RRV-YF (**Fig 7B**), while histological analysis of quadriceps samples at day 10 pi showed much more prominent lesions in RRV-wt than RRV-YF infected mice (**Fig 7C and S6A Fig**). In addition, viraemia for RRV-YF was slightly reduced compared to RRV-wt, reaching significance at 2 days pi, which corresponded to the viraemia peak (**Fig 7D**). Equally, although not significant, a trend suggesting lower viral titres at day 1 pi in the spleen, quadriceps and ankle, and at 6 and 10 days pi in quadriceps and ankle was observed (**Fig 7E**). Taken together, these results indicate that a mutation in the YXXM motif that reduces AKT hyperactivation results in attenuated infection compared to RRV-wt, and that AKT activation contributes to pathogenesis.

## Discussion

The importance of cellular energy metabolism in determining the outcome of a viral infection is increasingly recognised. However, the impact of a sustained metabolic activation on viral pathogenesis remains unclear. Primarily using SFV as a model alphavirus, we show changes in central metabolism that accompany viral infection, reveal the molecular mechanisms responsible for these changes, and, for the first time, explore their impact on viral pathogenesis *in vivo* using the relevant human pathogen RRV.

Using a combination of ^1^H- and ^13^C-NMR spectroscopy and GC-MS, we show that SFV infection increases and redirects glucose metabolism into macromolecular synthesis, by activating both glycolysis and the PPP. This metabolic switch leads to higher synthesis of nucleotides and fatty acids, critical building blocks for the formation of new SFV virions. Indeed, pharmacological inhibition of either of these pathways during virus replication results in reduced viral titres. As previously shown for cancer cells, and more recently for a number of viruses [24], increased glycolysis maximises the processing of glucose into macromolecules, and this provides a higher proliferative advantage than generation of ATP alone. Our work further emphasises the role of the PPP, which not only generates nucleotides for viral nucleic acid synthesis, but also contributes to lipid synthesis by providing NADPH. Interestingly, the kinetics of the metabolic shift observed in SFV infected cells mirrors the kinetics of viral replication, suggesting the importance of continued synthesis of metabolic intermediates during the production of new virions.

Mechanistically, we discovered that the metabolic changes induced by SFV infection are triggered through hyperactivation of the PI3K/AKT pathway. Moreover, we showed that a YXXM motif in SFV nsP3 is responsible for this activation through interaction with p85, the regulatory subunit of PI3K. Mutations in this motif in SFV nsP3 abolish hyperactivation of the pathway and lead to a metabolic profile consistent with increased consumption of metabolic intermediates, in the absence of a major increase in new metabolic synthesis. Moreover, fewer infectious particles are released upon infection with the SFV-YF mutant, suggesting that activation of glucose metabolism is important for maximal viral replication. In this study, AKT activation was seen in a variety of mammalian cells, including human SH-SY5Y, murine C2C12 and MEFs (upon transfection with Myr-Pal-nsP3), BHKs, and rat primary neurons, suggesting that this mechanism is not cell-specific and is conserved in different mammalian species. Whether the same is true in arthropod vectors and whether it represents an advantage for viral replication in the different species involved in virus transmission remains an interesting question. SINV, a related alphavirus that lacks the YXXM motif, has been shown to replicate largely independently of the PI3K/AKT/mTOR pathway in human cells [25], but to activate the pathway to some extent in arthropod cells [26]. We show that infection of differentiated SH-SY5Y cells with SINV does not lead to detectable activation of AKT, increases cellular glycolysis to a much smaller extent than SFV and does not alter fatty acids levels. This suggests that these two viruses have evolved distinct mechanisms for regulating lipid synthesis in mammalian cells, but are both dependent on glycolysis and the PPP.

Sequence analysis of nsP3 of all alphaviruses shows that YXXM motifs are present in nsP3 of SFV, RRV and also Getah virus and Sagiyama virus, which are equine pathogens in Asia [27], as well as Middelburg virus, which mainly infects sheep, goats and horses in Africa [28]. We found the YXXM motif to be highly conserved in nearly all the sequenced clinical isolates of the human pathogen RRV, despite its localisation within the hypervariable region of nsP3, in which sequence features are poorly conserved. Consistent with a critical role for AKT activation, mice infected with RRV-YF showed lower viraemia at the infection peak (2 days pi) and milder disease outcome compared with RRV-wt. Interestingly, failure to activate AKT *in vivo* attenuates viral replication even in the presence of an additional regulatory mechanism that appears to activate both glucose and glutamine metabolism. It is likely that while a compensatory mechanism is sufficient to maintain equal levels of viral replication *in vitro*, AKT activation is required for maximal production of new viral progeny *in vivo*, likely through increased fatty acids synthesis. It is possible that the trend of lower levels of viral replication is also responsible for a less sustained and aggressive immune activation, as indicated by lower levels of IFN-β in the lymph nodes and spleen (**S6B Fig**) and, as a consequence, milder disease progression after viral clearance (day 6 pi).

Intriguingly, while not conserved in all alphaviruses, activation of AKT via a YXXM motif has been reported for viruses in different families, including influenza virus [29] and herpes simplex virus [30], suggesting that different viral pathogens have evolved to use the same cellular mechanism of PI3K activation. While a number of viruses have been shown to activate PI3K, this is the first study that demonstrates a link between virus-induced activation of PI3K, cellular energy metabolism, viral replication, and *in vivo* pathogenesis, and explores the conservation of this mechanism of metabolic activation across different members of the alphavirus family.

As an association between high blood virus titres and the severity of arboviral infections has been demonstrated [31], inhibition of virus-induced pathways of metabolic regulation may be a viable antiviral strategy. If rapidly replicating viruses are hyper-dependent on generating new biomass, inhibition of the pathways responsible for metabolic activity might suffice to reduce viral titres to non-pathogenic levels without compromising the basal metabolic activity of the host. This scenario is particularly attractive because, by targeting common pathways of metabolic activation, a single drug or cocktail of drugs might be used to target a wide range of viral pathogens. Additionally, this study also reveals that even closely related viruses may develop different or redundant mechanisms of metabolic activation. Whether these differences arise from or are responsible for differences in virus replication or tropism, or what their impact is on the development of broad-spectrum antivirals remain compelling questions calling for a systematic study of the metabolic changes induced by different viruses and of the signalling pathways involved.

## Materials and methods

### Ethics statement

All animal experiments were approved by the Animal Ethics Committee of Griffith University (Gly/01/14/AEC). All procedures conformed to the National Health and Medical Research Council of Australia.

### Cells

SH-SY5Y cells (ATCC CRL-2266) were grown in 45% F-12 media and 45% DMEM with 10% (v/v) foetal calf serum (FCS, PAA), 1X non-essential amino acids, 1X sodium pyruvate, 20 mM HEPES, 1 mM L-glutamine, and 1X penicillin/streptomycin (p/s) (all from Gibco). For differentiation, 10 μM retinoic acid (Calbiochem) was added to the culture medium every 2–3 days for 6 days. C2C12 cells (ATCC CRL-1772) were grown in 10% DMEM GlutaMAX with 10% (v/v) FCS. For differentiation cells were plated on collagen coated dishes and cultured with DMEM GlutaMAX supplemented with 10% Horse Serum (Sigma) for 5 days. Immortalized mouse embryonic fibroblasts (MEF, kind gift from Nancy Kedersha, Harvard Medical School) and HEK293 cells (ATCC) were grown in DMEM, 10% (v/v) FBS, 2 mM L-glutamine, and 1X p/s. BHK-21 [C-13] (ATCC CCL-10) were grown in GMEM supplemented with 10% (v/v) FCS, 10% (v/v) tryptose phosphate broth (TPB, Gibco), and 1X p/s. Rat cortical neurons were isolated from P0 pups. Cortices were dissected in HBSS (Gibco) and trypsinized for 30 min at 37°C. Cells were collected by centrifugation, resuspended in Neurobasal media (ThermoFisher Scientific) supplemented with B27 (Gibco), triturated, and plated on glass coverslips coated with 1mg/ml poly-L-lysine (Sigma Aldrich). Neurons were incubated at 37°C with 5% CO_2_ for 3 days. For nutrient and growth factor depletion (starvation), cells were treated with Earle’s balanced salt solution (EBSS; Sigma). as previously described [32].

### Viruses

SFV and SFV-GFP (gift of Dr Giuseppe Balistreri, University of Helsinki) were expanded and titrated on BHK-21 cells. The stock used for the metabolomic experiments was concentrated and purified by centrifugation through a sucrose cushion. The SFV-Δ50 mutant was described previously [33]. SFV-wt, SFV-YA, and SFV-YF were rescued by transfection of BHK-21 cells with the infectious plasmid pCMV-SFV4 [34] and harvesting the cell culture supernatant upon appearance of cytopathic effect as described [16]. The mutations described in the text were introduced using Gibson assembly technology (NEBuilder HiFi DNA Assembly Master Mix, New England BioLabs, according to the manufacturer’s instructions) and verified by sequencing. SINV (kind gift of Penny Powell, University of East Anglia) was expanded and titrated in BHK-21 cells. Wild type virulent RRV (strain T48, here referred to as RRV-wt) was rescued from pRR64 [35] as described [36]. To generate RRV-YF, the codon for tyrosine 356 of RRV nsP3 was mutated to phenylalanine in pRR64 as above.

### Plasmids and transfection

Construction of Myr-Pal-nsP3-wt-FLAG was described previously [16]. To generate the Myr-Pal-nsP3-YF-FLAG, the nsP3-wt sequence was replaced with that of nsP3-YF, amplified from pCMV-SFV4-YF by PCR. Plasmid pEBB/PP-nsP3-wt, encoding SFV nsP3 with a biotin acceptor peptide [37] was used as a control without Myr-Pal signal. For p85, the following expression plasmids were employed: pEYFP-p85-α and the mutant pEYFP-p85-α-RARA [19] (kind gift of Ji Luo [National Cancer Institute, USA]); EYFP indicates the enhanced yellow-fluorescent protein); pEYFP-C1-p85-β (Addgene plasmid # 1408) and empty vector pEGFP-C1 (Clontech, EGFP indicating the enhanced green-fluorescent protein). All plasmids were verified by sequencing (Eurofins). Cells were transiently transfected using Lipofectamine 2000 (Life Technologies) according to the manufacturer’s instructions.

### ^1^H-NMR spectroscopy and GC-MS

Metabolomic analysis was performed as previously described [8,38,39] and as more extensively outlined in the Supplementary information.

### Virus release assays (plaque assay)

Differentiated SH-SY5Y cells were infected with SFV at MOI 3 in complete media with the indicated concentrations of inhibitors. Sixteen hours later, media was harvested and 500 μl of 10-fold serial dilutions of media were used to infect monolayers of BHK-21 cells for 2 h at 37°C. Infectious media were then replaced by a semisolid carboxymethylcellulose overlay (Rectapur, low viscosity, VWR; 1.5% in MEM). 48 h later the overlay was removed, cells washed in PBS, and plaques revealed by crystal violet staining. 2DG, DHEA, and Wortmannin were purchased from Sigma Aldrich, LY294002 from Cell Signaling Technology, and MK-2206 from Selleckchem.

### Infectivity assay

SH-SY5Y cells were seeded in 96 well format, differentiated and infected with SFV-GFP in culture media containing the indicated dilutions of inhibitors (MOI 3). 8 h later cells were washed and fixed in 4% formaldehyde, and the nuclei stained with Hoechst (5 μg/ml ThermoFisher Scientific). Images were acquired using an Opera high-content spinning-disk confocal microscope (PerkinElmer) and the percentage of infected cells quantified using the Columbus Image Data Management and Analysis Software (PerkinElmer).

### Cell lysis and immunoprecipitation

Cells were washed with PBS prior to lysis in lysis buffer (20 mM 4-(2-hydroxyethyl)-1-piperazineethanesulfonic acid [HEPES], pH 7.4, 110 mM potassium acetate, 2 mM magnesium chloride, 0.1% [vol/vol] Tween 20, 1% [vol/vol] Triton X-100, 0.5% [wt/vol] sodium deoxycholate, and 500 mM sodium chloride), supplemented with Complete protease inhibitor and PhosSTOP phosphatase inhibitor cocktails (Roche) on ice. After centrifugation (20,000×g for 10 min, 4°C), cell lysates were incubated with mouse-anti-GFP (Abcam, ab1218) or rabbit-anti-p85 (Cell Signaling Technology, catalog numbers 4257 and 4292, used at 1:1 ratio) antibodies for 20 min at room temperature by using a rotator, followed by incubation with protein G magnetic beads (GE Healthcare) at 4°C overnight. Immunoprecipitation samples were washed with lysis buffer, eluted with 4× reducing NuPAGE LDS sample buffer (Life Technologies), heated at 95°C for 5 min and analyzed by SDS-PAGE and western blotting.

### Immunoblots, immunofluorescence, and RT-PCR

Detailed procedure, antibodies, and primers are listed in the supporting file.

### Mouse infections, disease monitoring and histology

C57BL/6 mice were obtained from the Animal Resources Centre (Perth, Australia). Twenty-day old C57BL/6 mice were inoculated subcutaneously (s.c.) in the thorax below the right fore limb with 10^4^ PFU of RRV-wt or RRV-YF diluted in PBS to a volume of 50 μL. Mock-infected mice were inoculated with PBS only. Mice were weighed and scored for disease daily. RRV disease scores were assessed based on strength and hind-leg dysfunction using the scale described in the supplementary file. Mouse quadriceps were collected and fixed in 4% paraformaldehyde (PFA), followed by paraffin embedding. Samples were cut into 5 μm-thick sections and stained with hematoxylin and eosin. Images were taken using a Nikon microscope.

## Supporting information

S1 Fig **A.** Brightfield images showing the morphological difference between non-differentiated (left) vs differentiated SH-SY5Y, after 5 days treatment with 10 μM retinoic acid (right). Scale bar = 55 μm. **B**. Immunofluorescence staining showing accumulation of the SFV envelope proteins E1-E2 (in red) at the indicated time-points, as a marker of virus infection and replication in differentiated SH-SY5Y. Infection of all cells becomes clear at 8h (MOI 5). Blue: nuclei. Scale bar = 10 μm. Concentrations (mM) at the indicated time points of lactate and glucose in the media (**C**), and lactate, glucose and glycerophosphocholine in cells (**D**) following infection with SFV at MOI 10 or mock infection. **E.** Concentration (mM) of glycerophosphocholine in SFV-infected and control cells, after 8 hours in the presence of [U-^13^C]glucose, and concentrations (mM) of succinate and AMP in SFV-infected and control cells in parallel control experiments performed with unlabelled glucose. Six samples per group were analysed. Data are presented as means ± SD. **F.** Cell viability upon treatment with 25 mM 2DG or 100 μM DHEA. MTT assay was performed after 16 h treatment. Viability is relative to untreated cells. Data are presented as means ± SEM. **G.** Concentrations (mM) of lactate and glucose in the media (left hand panel) or lactate, glucose and AMP in the cells (middle and right hand panels) in differentiated SH-SY5Y cells treated for 16h with 25 mM 2DG or 100 μM DHEA. Data are presented as mean ± SD. **H.** SH-SY5Y were infected with SFV-GFP at MOI 3 and at the same time treated with the indicated concentrations of 2DG (top) or DHEA (bottom). Cells were fixed 8h later and infected cells counted by microscopy. Data are expressed as percentages of inhibition relative to untreated controls. The red square highlights the concentration used in all other experiments.* 0.05> p < 0.01; ** 0.01> p < 0.001; *** p < 0.001. Statistics as in Fig 1.
(TIF)

S2 Fig **A. Left side panels:** Kinetics of phospho-AKT (S473) activation (in red, left), or of neuronal beta III tubulin (in green) and nuclei (in blue) (right) in primary rat cortical neurons infected with SFV (MOI 5). Mock-infected samples were harvested at 8 hpi and the positive control (+ve) was obtained by 20 mins treatment with 200 μM hydrogen peroxide and 100 μM sodium orthovanadate. Scale bar = 15 μm. **Right side panels:** Immunofluorescence staining showing accumulation of the SFV envelope proteins E1-E2 (in red) at different time-points, as a marker of virus infection and replication in rat primary cortical neurons. Infection of all cells becomes clear at 8h (MOI 5). Green: anti beta-III tubulin; Blue: nuclei. Representative pictures are shown. **B.** Quantitative analysis of the experiment illustrated in A. The graph displays the area positive for P-AKT (S473) staining for each condition, normalised by the number of cells in the field. **C.** Real time quantitative PCR analysis showing transcription of the indicated glycolytic genes at different times after SFV infection of differentiated SH-SY5Y. Data are shown as fold induction over mock-infected cells and represent mean values ± SEM of three replicates. **D.** Cell viability upon treatment with indicated concentrations of Wortmannin or 50 μM LY294002. MTT assay was performed after 16 h treatment. Viability is relative to untreated cells. Data are presented as means ± SEM. **E.** SH-SY5Y were infected with SFV-GFP at MOI 3 and at the same time treated with the indicated concentrations of Wortmannin. Cells were fixed 8h later and infected cells were counted by microscopy. Data are expressed as percentages of inhibition relative to untreated controls. The red square highlights the highest concentration used in other experiments. **F.** Synthesis of new virions from SH-SY5Y infected with SFV at the same time or 2 h before treatment with 2 μM Wortmannin. **G**. Synthesis of new virions from SFV-infected SH-SY5Y **(top)** or rat primary cortical neurons **(bottom)** after treatment with 50 μM of the PI3K inhibitor LY294002, administered at the same time as SFV infection (MOI 3). After 16h, virions in the supernatant were quantified by plaque assay. Data are presented as means ± SEM. Statistics as in Fig 1.
(TIF)

S3 Fig **A.** Schematic showing the organisation of nsP3, highlighting the position of the YXXM motif in the C-terminus. **B.** Localisation of SFV-wt and SFV-YF replication complexes at 8 hpi (MOI 10), showing dsRNA (white) and nuclei (DRAQ5, blue). Representative confocal micrographs are shown, scale bar = 10 μm. **C.** Western blot analysis of lysates from cells infected for 8 h at MOI 10 with the indicated viruses, together with densitometry of phosphorylated AKT (S743), calculated as described in Fig 3A. **D.** Model of SFV nsP3-mediated PI3K activation.
(TIF)

S4 Fig **A.** Kinetics of AKT activation in SH-SY5Y cells infected with WT SFV and SFV-YF at MOI 5. Mock-infected sample was harvested at 8 h. Positive control as in Fig 5. **B.** Growth curves of WT SFV and SFV-YF in SH-SY5Y cells infected at MOI 0.1. At the indicated time-points, media was harvested and titrated by plaque assay. Data are presented as means ± SEM. Concentration (mM) of **C.** glutamate and glutamine (MOI 5), **D.** succinate (MOI 5), and **E.** glutamine and glutamate (MOI 1) in SFV- (black bars) or SFV-YF- (gray bars) infected cells, and in control cells (white bars). Six samples per group were analysed. Data are presented as means ± SD. **F.** HOS cells were infected with WT SFV or SFV-YF (MOI of 10 for 1 h) or mock infected and then supplemented with complete medium (no starvation) or EBSS (starvation) prior to lysis and western blot analysis for the indicated proteins. **G.** HOS cells were infected with WT SFV at MOI 10 in the presence or absence of rapamycin, and virus titres at each time point were determined by plaque assay. **H.** Cell viability upon treatment with 7 μM MK-2206. MTT assay was performed after 16 h treatment. Viability is relative to untreated cells. Statistics as in Fig 1.
(TIF)

S5 Fig **A.** Alignment of the C-terminal regions of SFV-nsP3 (GenBank accession number AKC01667) and RRV-nsP3 (UniProtKB/Swiss-Prot: P13888.1). The YXXM motif is shown in green. **B.** AKT activation in BHK cells infected with RRV-wt or RRV-YF at MOI 5 and lysed at 8 hpi. **C.** Replication complexes localization in BHK cells infected with RRV-WT or RRV-YF T MOI 5 and fixed at 8 hpi. Red: dsRNA, blue, nuclei stained with DRAQ5. Representative micrographs, scale bar: 10 μm. **D.** Growth curves of RRV-wt and RRV-YF in BHK cells infected at MOI 0.1 (left panel) or 5 (middle panel) and in differentiated C2C12 cells infected at MOI 5 (right panel) for the indicated times. Virus titres at each time point were determined by plaque assay. Data show the mean ± SEM of three replicates.
(TIF)

S6 Fig **A.** Additional histology (hematoxylin-eosin staining) of all mice sacrificed at day 10 pi. Scale bar: 100 μm. **B.** IFN-β levels in lymph nodes and spleen of mice mock-infected or infected with RRV-WT or RRV-YF at 1 day pi.
(TIF)

S1 Table Metabolites assigned by ^1^H-NMR in SH-SY5Y cells. In the first column the assigned metabolite for each signal is indicated, in the second the chemical shift, in the third the multiplicity. s = singlet, d = doublet, t = triplet, q = quadruplet, dd = doublet of doublets, m = multiplet, c = complex. In the last two column, the concentrations of each metabolites (mM) in Mock and SFV samples are shown. Metabolites listed in this table refer to the experiments illustrated in Fig 1.
(DOCX)

S2 Table Fatty acids identified by GC-MS in SH-SY5Y cells. In the first column the retention time (in minutes) is indicated, in the second the fatty acid associated to the peak, in the third and fourth the concentration of fatty acid in Mock and SFV samples, respectively. Metabolites listed in this table refer to the experiments illustrated in Fig 1.
(DOCX)

S1 Text Supporting material and methods. (DOCX)
