# Supplementary figures and images for: Alphavirus-induced hyperactivation of PI3K/AKT directs pro-viral metabolic changes
Source: PLoS Pathog. 2018 Jan 29;14(1):e1006835. doi: 10.1371/journal.ppat.1006835 (PMC5805360; doi:10.1371/journal.ppat.1006835)

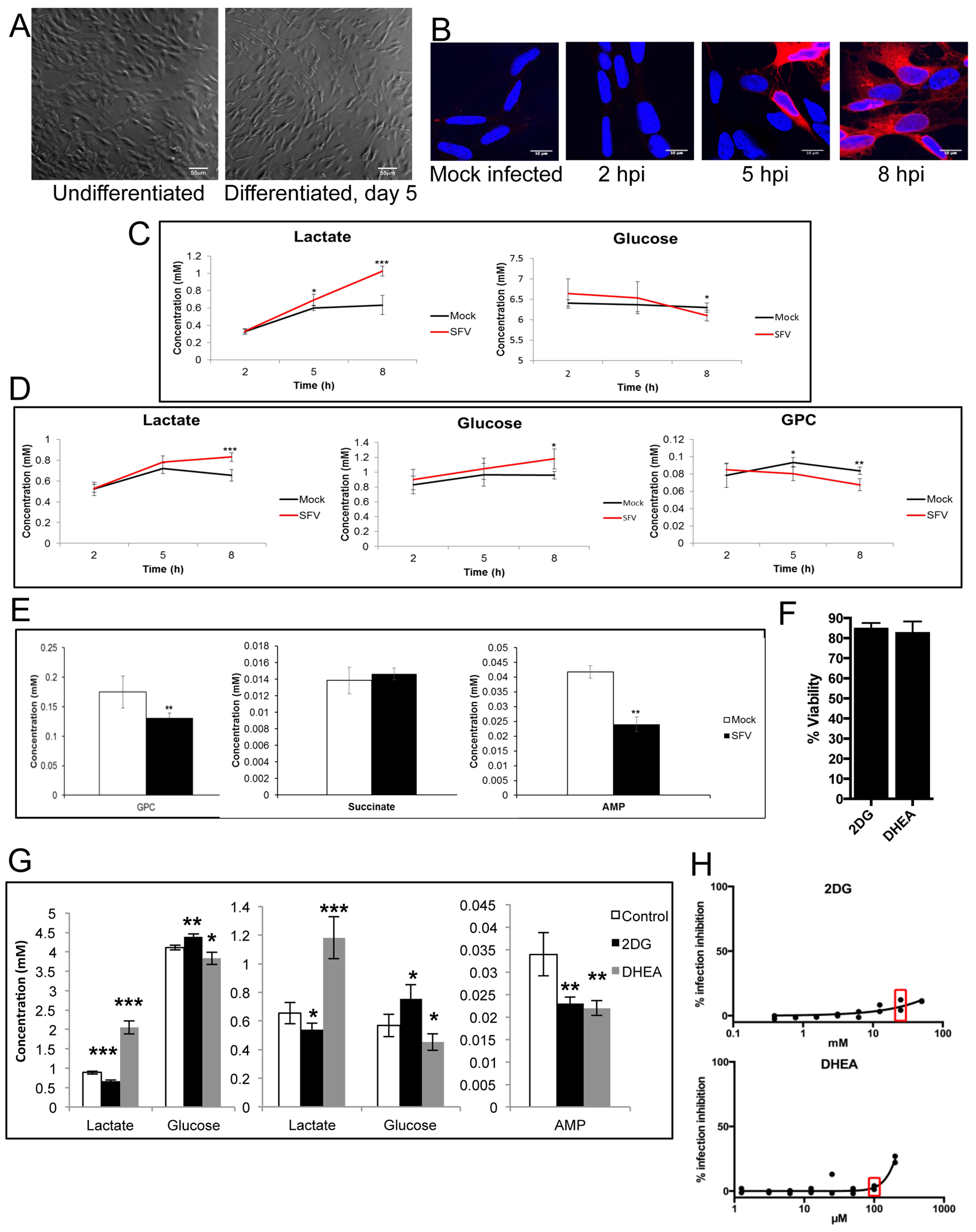

Supplement: S1 Fig — A. Brightfield images showing the morphological difference between non-differentiated (left) vs differentiated SH-SY5Y, after 5 days treatment with 10 μM retinoic acid (right). Scale bar = 55 μm. B. Immunofluorescence staining showing accumulation of the SFV envelope proteins E1-E2 (in red) at the indicated time-points, as a marker of virus infection and replication in differentiated SH-SY5Y. Infection of all cells becomes clear at 8h (MOI 5). Blue: nuclei. Scale bar = 10 μm. Concentrations (mM) at the indicated time points of lactate and glucose in the media (C), and lactate, glucose and glycerophosphocholine in cells (D) following infection with SFV at MOI 10 or mock infection. E. Concentration (mM) of glycerophosphocholine in SFV-infected and control cells, after 8 hours in the presence of [U-13C]glucose, and concentrations (mM) of succinate and AMP in SFV-infected and control cells in parallel control experiments performed with unlabelled glucose. Six samples per group were analysed. Data are presented as means ± SD. F. Cell viability upon treatment with 25 mM 2DG or 100 μM DHEA. MTT assay was performed after 16 h treatment. Viability is relative to untreated cells. Data are presented as means ± SEM. G. Concentrations (mM) of lactate and glucose in the media (left hand panel) or lactate, glucose and AMP in the cells (middle and right hand panels) in differentiated SH-SY5Y cells treated for 16h with 25 mM 2DG or 100 μM DHEA. Data are presented as mean ± SD. H. SH-SY5Y were infected with SFV-GFP at MOI 3 and at the same time treated with the indicated concentrations of 2DG (top) or DHEA (bottom). Cells were fixed 8h later and infected cells counted by microscopy. Data are expressed as percentages of inhibition relative to untreated controls. The red square highlights the concentration used in all other experiments.* 0.05> p < 0.01; ** 0.01> p < 0.001; *** p < 0.001. Statistics as in Fig 1. (TIF) [file ppat.1006835.s001.tif]

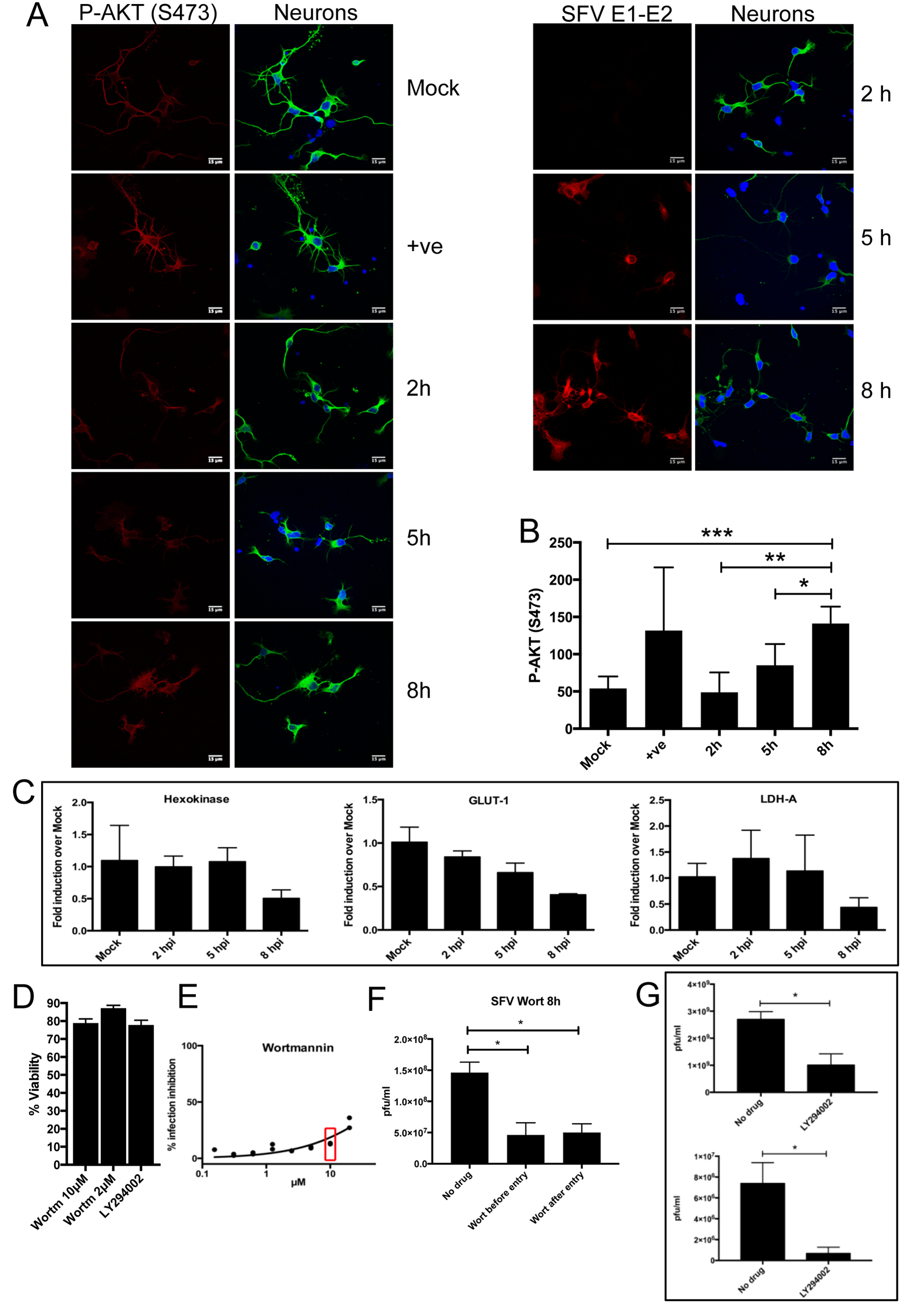

Supplement: S2 Fig — A. Left side panels: Kinetics of phospho-AKT (S473) activation (in red, left), or of neuronal beta III tubulin (in green) and nuclei (in blue) (right) in primary rat cortical neurons infected with SFV (MOI 5). Mock-infected samples were harvested at 8 hpi and the positive control (+ve) was obtained by 20 mins treatment with 200 μM hydrogen peroxide and 100 μM sodium orthovanadate. Scale bar = 15 μm. Right side panels: Immunofluorescence staining showing accumulation of the SFV envelope proteins E1-E2 (in red) at different time-points, as a marker of virus infection and replication in rat primary cortical neurons. Infection of all cells becomes clear at 8h (MOI 5). Green: anti beta-III tubulin; Blue: nuclei. Representative pictures are shown. B. Quantitative analysis of the experiment illustrated in A. The graph displays the area positive for P-AKT (S473) staining for each condition, normalised by the number of cells in the field. C. Real time quantitative PCR analysis showing transcription of the indicated glycolytic genes at different times after SFV infection of differentiated SH-SY5Y. Data are shown as fold induction over mock-infected cells and represent mean values ± SEM of three replicates. D. Cell viability upon treatment with indicated concentrations of Wortmannin or 50 μM LY294002. MTT assay was performed after 16 h treatment. Viability is relative to untreated cells. Data are presented as means ± SEM. E. SH-SY5Y were infected with SFV-GFP at MOI 3 and at the same time treated with the indicated concentrations of Wortmannin. Cells were fixed 8h later and infected cells were counted by microscopy. Data are expressed as percentages of inhibition relative to untreated controls. The red square highlights the highest concentration used in other experiments. F. Synthesis of new virions from SH-SY5Y infected with SFV at the same time or 2 h before treatment with 2 μM Wortmannin. G. Synthesis of new virions from SFV-infected SH-SY5Y (top) or rat primary cortical ne [file ppat.1006835.s002.tif]

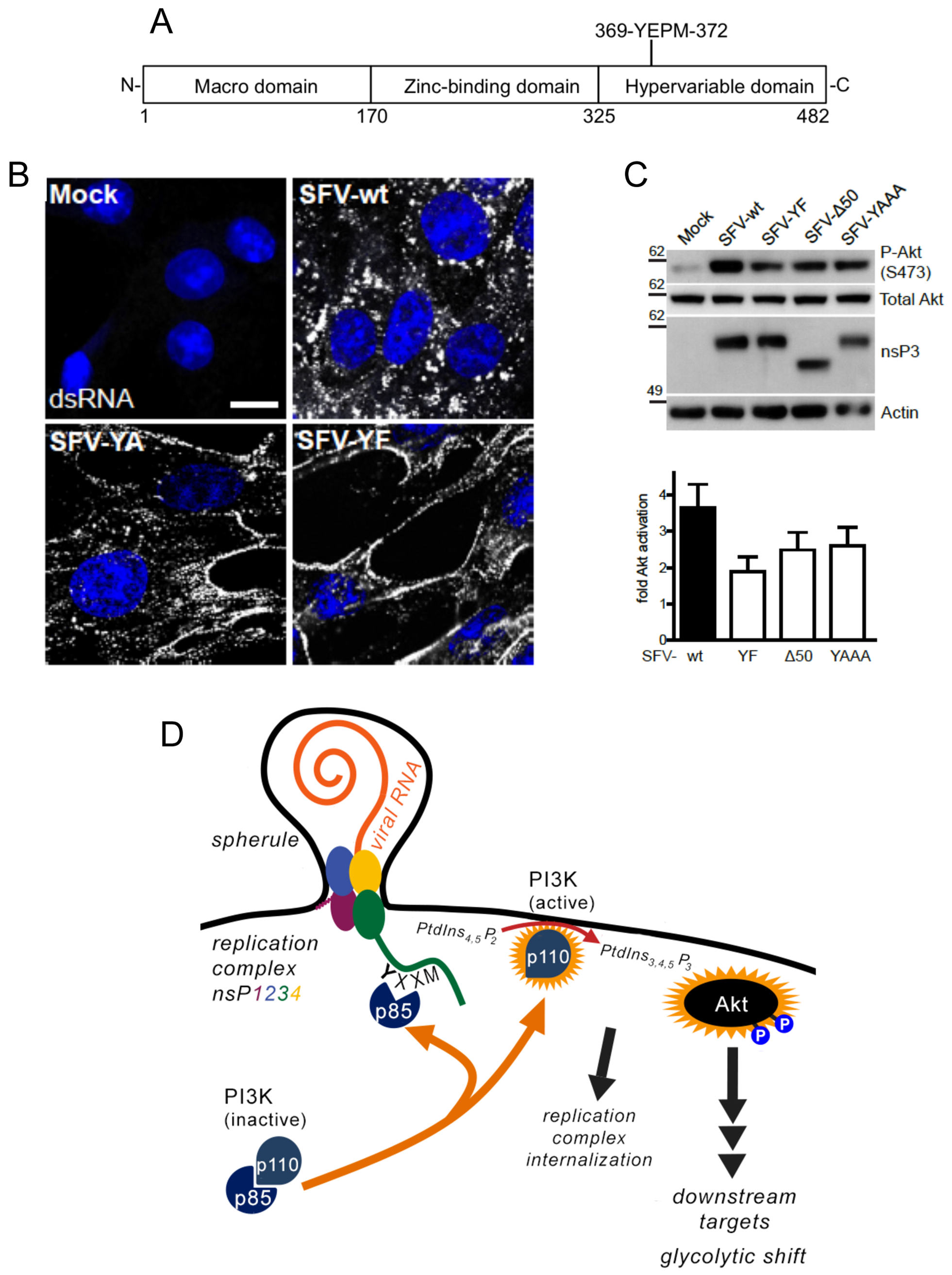

Supplement: S3 Fig — A. Schematic showing the organisation of nsP3, highlighting the position of the YXXM motif in the C-terminus. B. Localisation of SFV-wt and SFV-YF replication complexes at 8 hpi (MOI 10), showing dsRNA (white) and nuclei (DRAQ5, blue). Representative confocal micrographs are shown, scale bar = 10 μm. C. Western blot analysis of lysates from cells infected for 8 h at MOI 10 with the indicated viruses, together with densitometry of phosphorylated AKT (S743), calculated as described in Fig 3A. D. Model of SFV nsP3-mediated PI3K activation. (TIF) [file ppat.1006835.s003.tif]

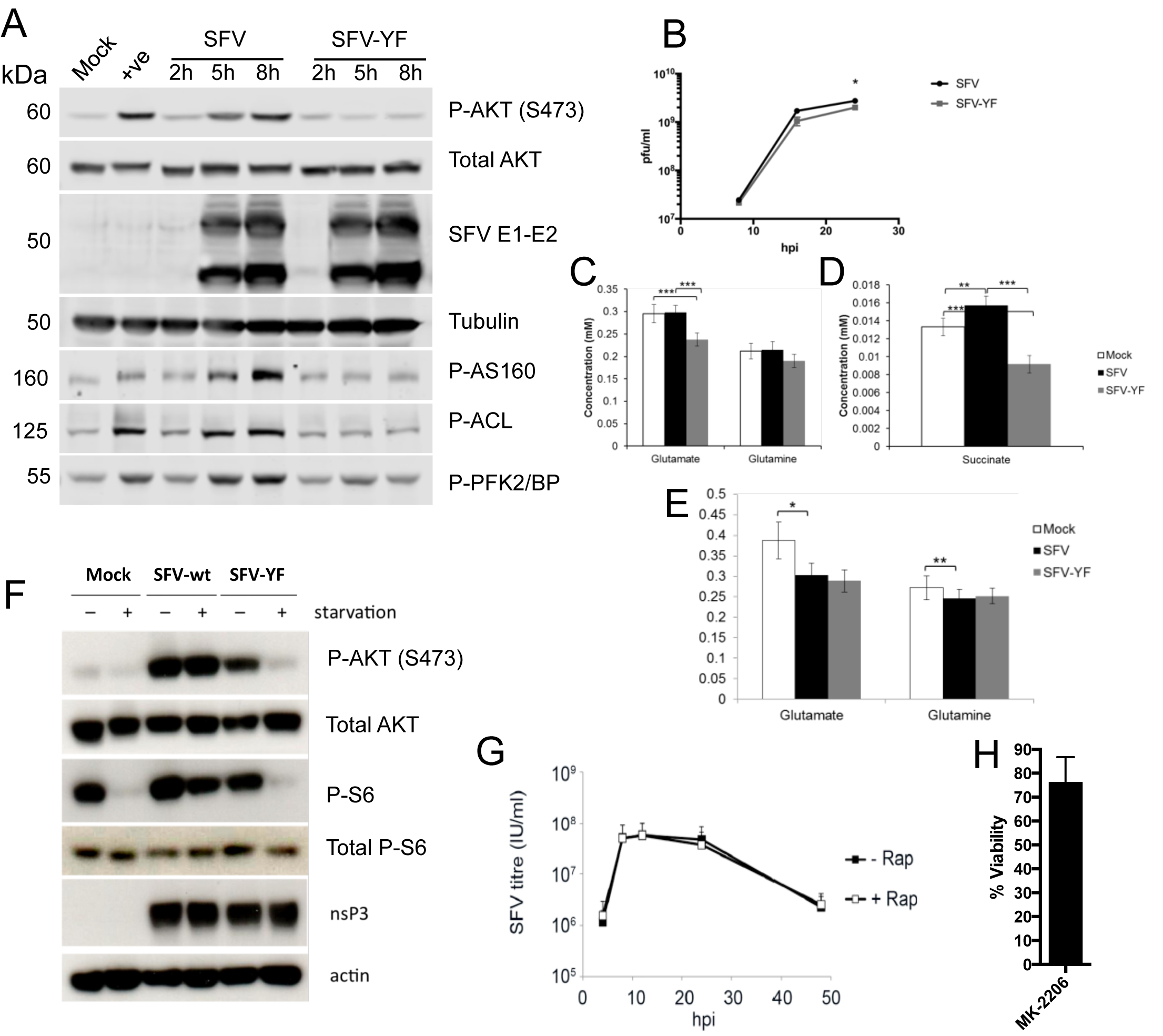

Supplement: S4 Fig — A. Kinetics of AKT activation in SH-SY5Y cells infected with WT SFV and SFV-YF at MOI 5. Mock-infected sample was harvested at 8 h. Positive control as in Fig 5. B. Growth curves of WT SFV and SFV-YF in SH-SY5Y cells infected at MOI 0.1. At the indicated time-points, media was harvested and titrated by plaque assay. Data are presented as means ± SEM. Concentration (mM) of C. glutamate and glutamine (MOI 5), D. succinate (MOI 5), and E. glutamine and glutamate (MOI 1) in SFV- (black bars) or SFV-YF- (gray bars) infected cells, and in control cells (white bars). Six samples per group were analysed. Data are presented as means ± SD. F. HOS cells were infected with WT SFV or SFV-YF (MOI of 10 for 1 h) or mock infected and then supplemented with complete medium (no starvation) or EBSS (starvation) prior to lysis and western blot analysis for the indicated proteins. G. HOS cells were infected with WT SFV at MOI 10 in the presence or absence of rapamycin, and virus titres at each time point were determined by plaque assay. H. Cell viability upon treatment with 7 μM MK-2206. MTT assay was performed after 16 h treatment. Viability is relative to untreated cells. Statistics as in Fig 1. (TIF) [file ppat.1006835.s004.tif]

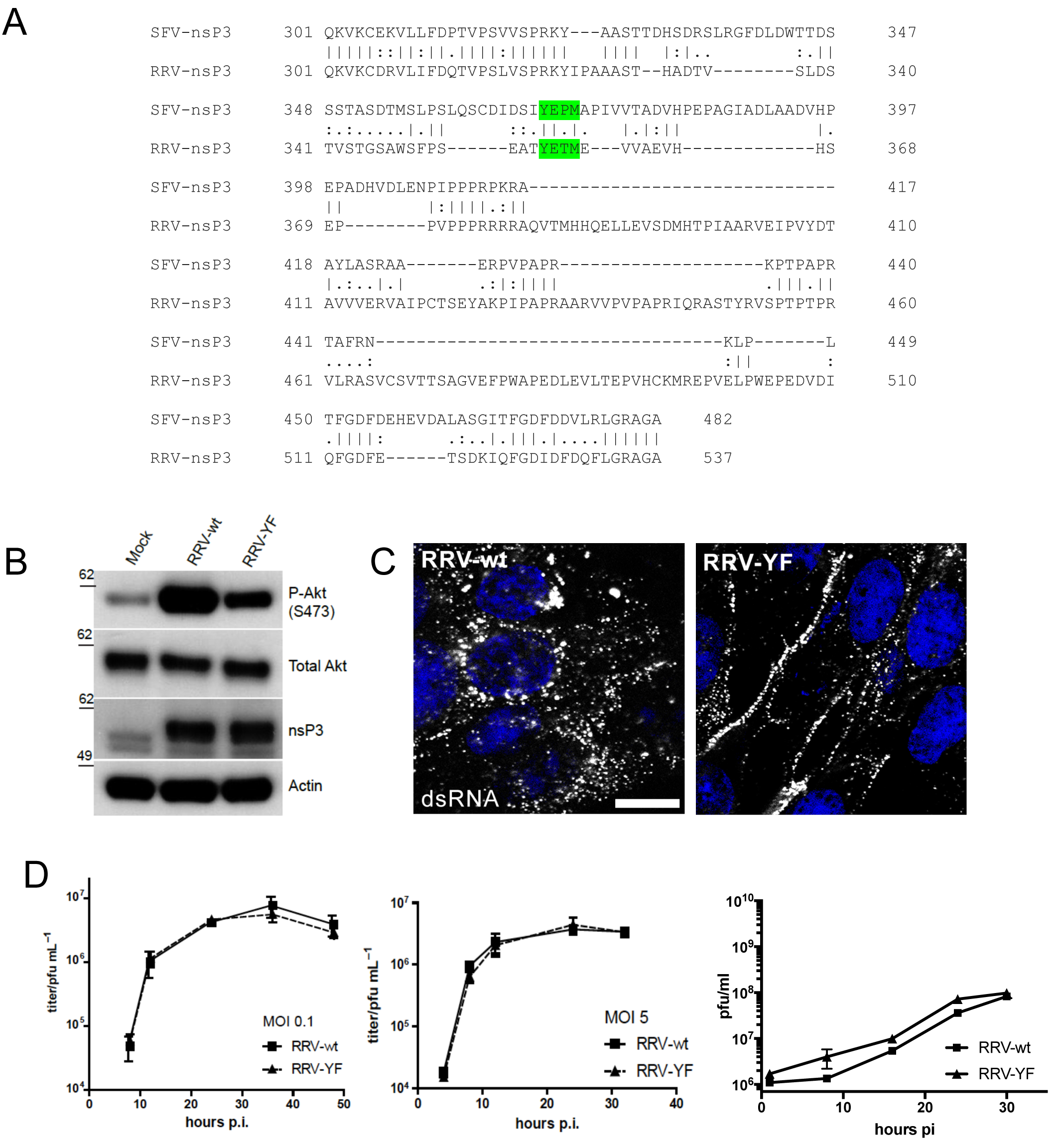

Supplement: S5 Fig — A. Alignment of the C-terminal regions of SFV-nsP3 (GenBank accession number AKC01667) and RRV-nsP3 (UniProtKB/Swiss-Prot: P13888.1). The YXXM motif is shown in green. B. AKT activation in BHK cells infected with RRV-wt or RRV-YF at MOI 5 and lysed at 8 hpi. C. Replication complexes localization in BHK cells infected with RRV-WT or RRV-YF T MOI 5 and fixed at 8 hpi. Red: dsRNA, blue, nuclei stained with DRAQ5. Representative micrographs, scale bar: 10 μm. D. Growth curves of RRV-wt and RRV-YF in BHK cells infected at MOI 0.1 (left panel) or 5 (middle panel) and in differentiated C2C12 cells infected at MOI 5 (right panel) for the indicated times. Virus titres at each time point were determined by plaque assay. Data show the mean ± SEM of three replicates. (TIF) [file ppat.1006835.s005.tif]

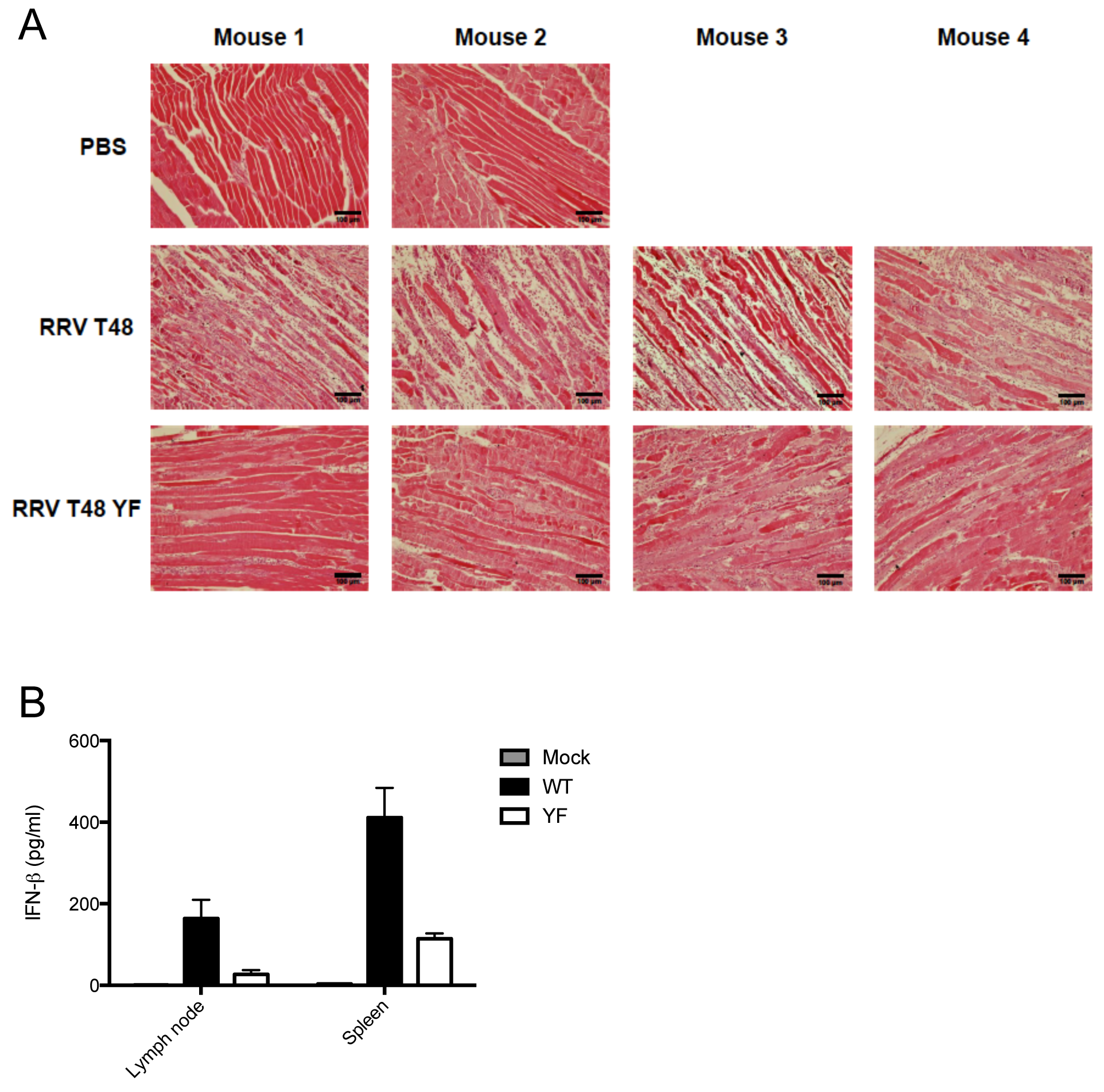

Supplement: S6 Fig — A. Additional histology (hematoxylin-eosin staining) of all mice sacrificed at day 10 pi. Scale bar: 100 μm. B. IFN-β levels in lymph nodes and spleen of mice mock-infected or infected with RRV-WT or RRV-YF at 1 day pi. (TIF) [file ppat.1006835.s006.tif]
