# Supplementary material for: Alphavirus-induced hyperactivation of PI3K/AKT directs pro-viral metabolic changes
Source: PLoS Pathog. 2018 Jan 29;14(1):e1006835. doi: 10.1371/journal.ppat.1006835 (PMC5805360; doi:10.1371/journal.ppat.1006835)
Supplement: S1 Table — In the first column the assigned metabolite for each signal is indicated, in the second the chemical shift, in the third the multiplicity. s = singlet, d = doublet, t = triplet, q = quadruplet, dd = doublet of doublets, m = multiplet, c = complex. In the last two column, the concentrations of each metabolites (mM) in Mock and SFV samples are shown. Metabolites listed in this table refer to the experiments illustrated in Fig 1. (DOCX) [file ppat.1006835.s007.docx]

| **Assignment** | **^1^H shift (δ)** | **Multiplicity** | **Mock (mM)** | **SFV (mM)** |
| --- | --- | --- | --- | --- |
| Isoleucine | 0.93 | t | 0.040±0.009 | 0.036±0.004 |
| Leucine | 0.94 | d | 0.050±0.007 | 0.041±0.006 |
| Leucine | 0.95 | d |  |  |
| Valine | 0.97 | d | 0.040±0.006 | 0.038±0.003 |
| Isoleucine | 0.99 | d |  |  |
| Valine | 1.02 | d |  |  |
| Isoleucine | 1.26 | m |  |  |
| Lactate | 1.31 | d | 0.51±0.05 | 0.73±0.08 |
| Alanine | 1.45 | d | 0.15±0.01 | 0.14±0.02 |
| Isoleucine | 1.47 | m |  |  |
| Leucine | 1.69 | m |  |  |
| Leucine | 1.73 | m |  |  |
| Acetate | 1.89 | s | 0.12±0.01 | 0.12±0.01 |
| Proline | 1.97 | m | 0.08±0.03 | 0.08±0.03 |
| Isoleucine | 1.96 | m |  |  |
| Proline | 2.02 | m |  |  |
| Glutamate | 2.03 | m | 0.15±0.01 | 0.14±0.01 |
| Proline | 2.06 | m |  |  |
| Glutamine | 2.08 | m | 0.07±0.01 | 0.08±0.02 |
| Glutamate | 2.12 | m |  |  |
| Glutamine | 2.14 | m |  |  |
| Valine | 2.26 | m |  |  |
| Glutamate | 2.32 | t |  |  |
| Proline | 2.34 | m |  |  |
| Glutamate | 2.36 | t |  |  |
| Malate | 2.37 | dd | 0.026±0.007 | 0.024±0.009 |
| Succinate | 2.4 | s | 0.018±0.006 | 0.015±0.006 |
| Glutamine | 2.42 | c |  |  |
| Citrate | 2.56 | d | 0.011±0.003 | 0.008±0.003 |
| Malate | 2.65 | dd |  |  |
| Citrate | 2.68 | d |  |  |
| Creatine | 3.03 | s | 0.018±0.002 | 0.013±0.001 |
| Tyrosine | 3.04 | dd | 0.017±0.003 | 0.015±0.002 |
| Phenylalanine | 3.11 | dd | 0.015±0.004 | 0.014±0.002 |
| Tyrosine | 3.18 | dd |  |  |
| Choline | 3.19 | s | 0.020±0.003 | 0.012±0.002 |
| Phosphocholine | 3.2 | s | 0.026±0.003 | 0.019±0.003 |
| sn-3-Glycerophosphocholine | 3.22 | s | 0.079±0.009 | 0.053±0.005 |
| Glucose | 3.23 | dd | 0.6±0.1 | 0.60±0.08 |
| Myo-Inositol | 3.27 | t | 0.05±0.01 | 0.04±0.01 |
| Phenylalanine | 3.28 | dd |  |  |
| Proline | 3.33 | m |  |  |
| Glucose | 3.39 | t |  |  |
| Glucose | 3.4 | t |  |  |
| Proline | 3.43 | m |  |  |
| Glucose | 3.45 | m |  |  |
| Glucose | 3.47 | t |  |  |
| Choline | 3.51 | m |  |  |
| Myo-Inositol | 3.52 | dd |  |  |
| Glucose | 3.54 | ddd |  |  |
| Phosphocholine | 3.57 | m |  |  |
| Valine | 3.61 | d |  |  |
| Myo-Inositol | 3.62 | t |  |  |
| sn-3-Glycerophosphocholine | 3.62 | dd |  |  |
| Isoleucine | 3.63 | d |  |  |
| sn-3-Glycerophosphocholine | 3.66 | m |  |  |
| sn-3-Glycerophosphocholine | 3.68 | dd |  |  |
| Glucose | 3.7 | t |  |  |
| Leucine | 3.72 | m |  |  |
| Glucose | 3.72 | dd |  |  |
| Glucose | 3.73 |  |  |  |
| Alanine | 3.74 | q |  |  |
| Glucose | 3.76 | m |  |  |
| Glucose | 3.8 | ddd |  |  |
| sn-3-Glycerophosphocholine | 3.86 | ddd |  |  |
| Glucose | 3.87 | m |  |  |
| Glucose | 3.9 | dd |  |  |
| Creatine | 3.91 | s |  |  |
| sn-3-Glycerophosphocholine | 3.91 | m |  |  |
| Tyrosine | 3.93 | dd |  |  |
| sn-3-Glycerophosphocholine | 3.94 | ddd |  |  |
| Phenylalanine | 3.99 | dd |  |  |
| Myo-Inositol | 4.05 | t |  |  |
| Choline | 4.06 | m |  |  |
| Lactate | 4.08 | q |  |  |
| Proline | 4.14 | m |  |  |
| Phosphocholine | 4.16 | m |  |  |
| Malate | 4.3 | dd |  |  |
| sn-3-Glycerophosphocholine | 4.31 | m |  |  |
| Glucose | 4.63 | d |  |  |
| Glucose | 5.22 | d |  |  |
| AMP | 6.14 | d | 0.025±0.006 | 0.016±0.004 |
| Tyrosine | 6.89 | d |  |  |
| Tyrosine | 7.19 | d |  |  |
| Phenylalanine | 7.32 | d |  |  |
| Phenylalanine | 7.35 | t |  |  |
| Phenylalanine | 7.41 | t |  |  |
| AMP | 8.27 | s |  |  |
| Formate | 8.45 | s | 0.037±0.002 | 0.042±0.008 |
| AMP | 8.5 | s |  |  |
