# Supplementary material for: Alphavirus-induced hyperactivation of PI3K/AKT directs pro-viral metabolic changes
Source: PLoS Pathog. 2018 Jan 29;14(1):e1006835. doi: 10.1371/journal.ppat.1006835 (PMC5805360; doi:10.1371/journal.ppat.1006835)
Supplement: S2 Table — In the first column the retention time (in minutes) is indicated, in the second the fatty acid associated to the peak, in the third and fourth the concentration of fatty acid in Mock and SFV samples, respectively. Metabolites listed in this table refer to the experiments illustrated in Fig 1. (DOCX) [file ppat.1006835.s008.docx]

| **RT (min)** | **Metabolite** | **Mock** | **SFV** |
| --- | --- | --- | --- |
| 8.27 | D-25 tridecanoic acid methyl ester C13:0 (IS) |  |  |
| 9.16 | Myristic acid methyl ester C14:0 | 0.030±0.002 | 0.036±0.002 |
| 9.98 | Pentadecanoic acid methyl ester C15:0 | 0.09±0.01 | 0.011±0.002 |
| 10.98 | Palmitic acid methyl ester C16:0 | 6.2±0.06 | 7.4±0.6 |
| 11.26 | Palmitoleic acid methyl ester C16:1 | 0.27±0.05 | 0.33±0.05 |
| 11.96 | Margaric acid methyl ester C17:0 | 0.16±0.01 | 0.19±0.02 |
| 13.2 | Stearic acid methyl ester C18:0 | 5.6±0.6 | 6.7±0.6 |
| 13.49 | Oleic acid methyl ester C18:1n9c | 4.8±0.8 | 5.4±0.6 |
| 13.56 | Vaccenic acid methyl ester C18:1n7c | 1.2±0.2 | 1.4±0.3 |
| 14.12 | Linoleic acid methyl ester C18:2n6 | 0.16±0.02 | 0.19±0.03 |
| 16 | 11-Eicosenoic acid methyl ester C20:1 | 0.16±0.01 | 0.18±0.02 |
| 16.47 | 11,14-Eicosadienoic acid methyl ester C20:2n6 | 0.25±0.04 | 0.26±0.03 |
| 17.2 | 8,11,14-Eicosatrienoic acid methyl ester C20:3n6 | 0.35±0.03 | 0.41±0.07 |
| 17.51 | Arachidonic acid methyl ester C20:4n6 | 2.2±0.2 | 2.5±0.1 |
| 18.51 | 5,8,11,14,17-Eicosapentenoic acid methyl ester C20:5n3 | 0.12±0.01 | 0.11±0.02 |
| 21.32 | 7,10,13,16,19-Docosapentaenoic acid methyl ester C22:5n3 | 0.59±0.05 | 0.59±0.06 |
| 21.6 | 4,7,10,13,16,19-Docosahexaenoic acid methyl ester C22:6n3 | 1.50±0.08 | 1.5±0.2 |
