# Supplementary material for: Alphavirus-induced hyperactivation of PI3K/AKT directs pro-viral metabolic changes
Source: PLoS Pathog. 2018 Jan 29;14(1):e1006835. doi: 10.1371/journal.ppat.1006835 (PMC5805360; doi:10.1371/journal.ppat.1006835)
Supplement: S1 Text — (DOCX) [file ppat.1006835.s009.docx]

**Supporting Information Materials and Methods**

**Cell growth.** Six confluent T175 flasks per conditions were used for NMR experiments on SH-SY5Y (7-10 million cells). Cells were infected at MOI 5 (SFV) or 500 (SINV) in 10 ml of culture media with 2% FCS. After the indicated time-points, 1ml of media was harvested, cleared of cells by centrifugation, and snap frozen in liquid N_2_. Cells were scraped in the remaining media, centrifuged and the pellet snap frozen to quench metabolic activity. For primary cortical neurons, 300,000 cells per samples were infected with SFV at MOI 10 in 200 ml of neurobasal media with B27 supplement. NMR studies on rat primary neurons were performed from media only. For labelling experiments the same protocol was followed, but the experiment was performed in glucose-free media supplemented either with 4500 mg/l of universally labelled glucose or with 4500 mg/l of non-labelled glucose.

**Metabolite extraction.** Metabolites were extracted from both cells and media using a methanol–chloroform–water (2:2:1) procedure. 600 μl of methanol–chloroform mix (2:1 v:v) were added to the cells and samples were sonicated for 15 min at room temperature. 200 μl each of chloroform and water were added, the samples centrifuged and the separated aqueous and lipid phases collected. The procedure was repeated twice, and the aqueous and lipid fractions from each extraction pooled. The aqueous layer was dried overnight in an evacuated centrifuge. The lipid fraction was left to dry overnight at room temperature.

**NMR analysis of aqueous extracts.** The dried aqueous fractions were rehydrated in 600 μl D_2_O, containing 0.05 mM sodium-3-(tri-methylsilyl)-2,2,3,3-tetradeuteriopropionate (TSP) (Cambridge Isotope Laboratories, MA, USA) as an internal standard. The samples were analysed using an AVANCE II+ NMR spectrometer operating at 500.13 MHz for the ^1^H frequency and 125.721 MHz for the ^13^C frequency (Bruker, Germany) using a 5 mm TXI probe. The instrument is equipped with TopSpin 3.2. Spectra were collected using a solvent suppression pulse sequence based on a one-dimensional nuclear Overhauser effect spectroscopy (NOESY) pulse sequence to saturate the residual 1H water signal (relaxation delay = 2 s, t1 increment = 3 us, mixing time = 150 ms, solvent presaturation applied during the relaxation time and the mixing time). One hundred and twenty-eight transients were collected into 16 K data points over a spectral width of 12 ppm at 27 ºC. In addition, representative samples of each data set were also examined by two-dimensional Correlation Spectroscopy (COSY), using a standard pulse sequence (cosygpprqf) and 0.5 s water presaturation during relaxation delay, 8 kHz spectral width, 2048 data points, 32 scans per increment, 512 increments. Assignment of the peaks was done using the COSY spectra in conjunction with reference to previous literature and databases and the Chenomx spectral database contained in Chenomx NMR Suite 7.7 (Chenomx, Alberta, Canada).

**^13^C-NMR.** The dried aqueous fractions were resuspended in 600 μl D_2_O, containing 0.05 mM TSP as internal standard for ^1^H-NMR and 0.5 mM [^13^C]formate (Isotec-Sigma Aldrich, St. Louis, MO, USA) for ^13^C-NMR. For the 1H-13C HSQC (heteronuclear single quantum coherence) experiments, spectra were acquired using the standard Bruker pulse sequence hsqcetgpsp.3 (phase-sensitive gradient edited-2D HSQC, using adiabatic pulses for inversion and refocusing and background signals effects). 2048 data points were acquired over 12 ppm spectral width (acquisition time 170 ms) in F2 dimension, using 64 scans with 2s delay. For spectral width of 180 ppm 256 increments were acquired in F1 (acquisition time 5.6 ms), that resulted in the total experimental time of ~10 h per sample.

**NMR data processing.** 1D-NMR spectra were processed using ACD one-dimensional NMR processor (vers. 12, ACD, Toronto, Canada). Free induction decays were Fourier transformed following multiplication by a line broadening of 1 Hz, and referenced to TSP at 0.0 ppm. Spectra were phased and baseline corrected manually. Each spectrum was integrated using 0.02 ppm integral regions between 0.5 and 4.3, and 4.7–8.5 ppm. The spectral region for each sample was scaled such that the total sum of integrals for each sample was equal. The integrals of the different metabolites were obtained using Chenomx.

HSQC spectra were processed using TopSpin and peaks were picked in a manual manner.

**GC-MS analysis of organic extracts.** 50 μl of D-25 tridecanoic acid (200 μM in chloroform), 650 μl of chloroform/methanol (1:1 v/v) and 125 μl BF_3_/methanol (Sigma-Aldrich) was added to 100 μl organic extract dissolved in chloroform/methanol (1:1 v/v) (half of the organic material extracted for each sample). The samples were then incubated at 80 ºC for 90 min. 500 μl H_2_O and 1 ml hexane were added and each vial mixed. The organic layer was evaporated to dryness before reconstitution in 100 μl hexane for analysis. Using a Trace GC Ultra coupled to a Trace DSQ II mass spectrometer (Thermo Scientific, Hemel Hempstead, UK), 4 μl of the derivatised organic metabolites were injected onto a TR-fatty acid methyl ester (FAME) stationary phase column (Thermo Electron; 30 m × 0.25 mm ID × 0.25 μm; 70% cyanopropyl polysilphenylene-siloxane) with a split ratio of 20. The injector temperature was 230°C and the helium carrier gas flow rate was 1.2 ml/min. The column temperature was 60°C for 2 min, increased by 15°C/min to 150°C, and then increased at a rate of 4°C/min to 230°C (transfer line = 240°C; ion source = 250°C, EI = 70 eV). The detector was turned on after 240 s, and full-scan spectra were collected using 3 scans/s over a range of 50–650 *m*/*z*. Peaks were assigned using Food Industry FAME Mix (Restek 6098) and Bacterial Acid Methyl Ester (BAME) Mix solution (Supelco 47080).

**GC-MS data processing.** GC–MS chromatograms were analysed using Xcalibur, version 2.0 (Thermo Fisher), integrating each peak individually. Peaks were normalised to the internal standard.

**Multivariate analysis of metabolite profiles.** The set of metabolic profiles obtained were analysed by multivariate analysis. Datasets were imported into SIMCA-P 12.0 (Umetrics, Umeå, Sweden) for processing using PCA and PLS-DA (a regression extension of PCA used for supervised classification). ^1^H NMR data were Pareto scaled, in which each variable was centred and multiplied by 1/(Sk)1/2 where Sk is the standard deviation of the variable. GC–MS data were scaled to unit variance by dividing each variable by 1/(S_k_).

**Immunoblots.** Cells from 10cm^2^ dishes were harvested in 150 μl of lysis buffer (25mM Tris-HCl pH 7.5, 100mM NaCl, 1% Triton X-100, 0.5% NP40, 1mM EDTA, 1mM EGTA, supplemented with protease inhibitor (Roche) and phosphatase inhibitor (Sigma) cocktails and nuclear fractions were precipitated by centrifugation. Supernatants were transferred to clean tubes and 6X reducing loading buffer added. Samples were boiled for 5 mins and protein separated by SDS-PAGE and transferred onto a PVDF membrane. Primary antibodies: goat-anti-actin (1616; Santa Cruz), rabbit-anti-GFP (Abcam, ab290), rabbit-anti-p85 (Cell Signaling Technology, catalog numbers 4257 and 4292, used at 1:1 ratio), rabbit antiserum raised against SFV nsP3. Phospho-AKT (S473, Cell Signaling Technology (CST 9271S), AKT (CST 9272S), phospho-AS160 (CST 8619S), phospho-ACL (CST 4331S), phospho-PFK2/BF (CST 13064S) were diluted 1:1000 in 5% BSA/TBS 0.1% tween. The anti-tubulin antibody (mouse anti-tubulin clone DM1A, ascites fluid, Sigma) was used at 1:5000; anti-SFV E1-E2 (Marsh and Bron, 1997) at 1:1000; anti-SINV E1 (mouse monoclonal, kind gift of Margaret Kielian) at 1:40; anti-alphavirus antibody (Santa Cruz Alphavirus Antibody (3581): sc-58088) was used to detect RRV in Figure 5 and was used at 1:1000. Secondary antibodies (Licor IRDye anti-mouse 800 and anti-rabbit 680) were used at 1:10,000. Blots were imaged using a Licor Odyssey infrared imager.

**Immunofluorescence.** Samples were washed in PBS and fixed in 4% PFA for 30 mins. PFA was quenched with 50 mM ammonium chloride for 20 mins and cells permeabilised for 10 mins in 0.1% Triton in 0.2% BSA/TBS. Primary antibodies were incubate for 2h and secondary antibodies for 1h. Primary antibodies: mouse-anti-FLAG tag M2 (F1804, Sigma) was used 1:500; phospho-AKT (S473, CST 9271S) was used at 1:25; anti-beta III tubulin (Abcam, ab107216) at 1:1000; anti SFV E1-E2 at 1:500; anti SINV E1 neat. Secondary antibodies: Anti-chicken Alexa Fluor 488 (Jackson), anti-rabbit Alexa Fluor 647 (ThermoFisher Scientific), anti-rabbit Alexa Fluor 488, anti-mouse Alexa Fluor 555 (Molecular Probes). Samples were washed and coverslips mounted in Mowiol. Confocal images were acquired with an inverted Leica TCS SP3 confocal microscope, 63x oil objective (NA 1.4) and LASAF software, and processed using Fiji to segment and measure the area per cell positive for Ph-AKT (S473). Epifluorescence images were taken with a Zeiss Axiovert 200M epifluorescence microscope and processed in Adobe Photoshop. For staining of viral RCs, cells were fixed with 3.7% formaldehyde in PBS at the indicated times post-infection (p.i.) and permeabilized with methanol as previously described [36]. Mouse-anti-dsRNA (1:200; English and Scientific Consulting) was used as primary antibody, and Alexa 555-conjugated donkey-anti-mouse-IgG (1:1,000) (Molecular Probes) as secondary antibody. Nuclei were stained with the DNA dye DRAQ5 (1:1,000; Biostatus). Samples were imaged by confocal laser scanning microscopy using a Leica TCS SP5 X microscope equipped with a supercontinuum pulsed white laser.

**MTT assay.** 16 h after the indicated treatments, cells were incubated for 2h at 37 C with 5mg/ml Thiazolyl Blue Tetrazolium Bromide (MTT, Sigma). Media was then removed, cells washed once in PBS, and a 1:1 mixture of DMSO:Isopropanol was added to the cells for 20 mins at 37 C. Supernatant was transferred in a clean plate before reading at 570 nm.

**Real time quantitative qPCR**. RNA was extracted from SH-SY5Y cells plated in 10 cm^2^ format. RNA was extracted using a Qiagen RNAesy Plus kit. 500ng of extracted RNA were reverse transcribed using Qiagen Quantitect reverse transcription kit, and cDNA was diluted 5 fold. 2μl cDNA were used per reaction (10% v/v) and amplified using ThermoFisher Scientific SYBR Green master mix in a Biorad CFX 96 thermocycler. Data were analysed using the Pfaffl method, which accounts for slight differences in primer efficacy, and are presented as fold-induction compared to mock infected samples.

Primers are as follow:

GLUT-1 Forward: GATTGGCTCCTTCTCTGTGG,

GLUT-1 Reverse: TCAAAGGACTTGCCCAGTTT;

Hexokinase 2: Forward: CTGCTGGAGGTCAAGAGGAG,

Hexokinase 2 Reverse: AAGGTCCAAGGCCAAGAAGT;

LDH-A Forward: TTGGTCCAGCGTAACGTGAAC,

LDH-A Reverse: CCAGGATGTGTAGCCTTTGAG;

18S RNA Forward: GTAACCCGTTGAACCCCATT,

18S RNA Reverse: CCATCCAATCGGTAGTAGCG.

Beta-actin Forward: AGGCACCAGGGCGTGAT

Beta-actin Reverse: GCCCACATAGGAATCCTTCTGAC

**RRV disease score scale:**

0, no disease signs;

1, ruffled fur;

2, very mild hind limb weakness;

3, mild hind limb weakness;

4, moderate hind limb weakness and dragging of hind limbs;

5, severe hind limb weakness/dragging;

6, complete loss of hind limb function;

7, moribund.

Humane end point was defined as mice scored to 7 in clinical disease or show weight loss of greater than 15 % of their starting weight [64].

**IFN-βELISA.** Mouse spleen and lymph nodes were harvested day 1 post infection, processed in 1ml 1xPBS and UV-inactivated for 1 h. 100 μl of each sample were used to carry out IFN-β assay. The amount of IFN-β from mouse tissues was measured using a commercial VeriKine^TM^Mouse IFN Beta ELISA Kit (PBL InterferonSource), according to the manufacturer's instructions.

**Statistical analysis.** Data for mouse weight and plaque assays were analysed using two-way analysis of variance (ANOVA) with Bonferroni post hoc test. Differences in mouse disease scores were analyzed using the non-parametric Mann-Whitney test. All statistical analyses were performed with GraphPad Prism software.

**Reference**

MARSH, M. & BRON, R. 1997. SFV infection in CHO cells: cell-type specific restrictions to productive virus entry at the cell surface. *Journal of Cell Science*, 110, 95-103.
